# The cost-effectiveness of COVID-19 vaccination program among age-groups children, adults, and elderly in Europe: A systematic review

**DOI:** 10.1016/j.jvacx.2024.100580

**Published:** 2024-11-08

**Authors:** T. Untung, R. Pandey, P. Johansson

**Affiliations:** School of Public Health and Community Medicine, University of Gothenburg, Göteborg, Sweden

**Keywords:** COVID-19, Vaccination, Children, Elderly, Adult, Cost-effectiveness analysis

## Abstract

**Objectives:**

To prepare for future epidemics, the experiences from the vaccination programs in the COVID-19 pandemic need to be collated. This systematic review synthesizes health economic evidence of COVID-19 vaccination programs in European countries comparing the target groups children, adults, and elderly, to study whether the Swedish vaccination strategy was justified on cost-effectiveness grounds.

**Method:**

A literature search using the PICOS (Population, Intervention, Control, Outcomes, Study design) convention was conducted in the databases Medline, Embase, PsycInfo, CINAHL, and Tuft CEA Registry, Cochrane and INAHTA in February 2023. The inclusion criteria were economic evaluations (S) comparing COVID-19 vaccination (I) in age-groups children, adult, and elderly European residents (P) with non-vaccinated European residents (C) in terms of cost per QALY, cost differences, and net monetary benefit (O). Hand-search was done on selected websites and in reference lists of included reports. Title/abstract screening, full-text screening, and quality assessment with the Swedish HTA agency checklist were performed by two researchers. The reporting follows the PRISMA 2020 recommendations.

**Results:**

The database search resulted in 5,720 reports, title/abstract screening yielded 162 reports and after full-text screening, four reports remained. Two studies comparing vaccination of adults and elderly with high and moderate study quality were included. No study was found on the children population. The economic evidence indicated that COVID-19 vaccination of the elderly is cost-effective when compared with vaccination of the adult group, but the transferability to Swedish circumstances was inconclusive due to differences in outcome and cost data between Sweden and the included studies’ settings.

**Conclusion:**

The common European COVID-19 vaccination policy that prioritized the elderly population was the cost-effective option in the reviewed studies. The lack of transferability to Sweden precludes a clear conclusion on the Swedish vaccination policy.

## Highlights

• COVID-19 vaccination is reported cost-effective for elderly populations.
• The vaccination policy chosen by most European countries seems adequate.
• No cost-effectiveness evidence found for vaccination of children.

## Introduction

COVID-19, caused by the virus SARS-CoV-2, was officially named by WHO (World Health Organization) on February 11, 2020 [1], [2]. The virus is highly contagious and potentially fatal, which led the WHO to declare a global pandemic on March 11, 2020 [3]. European countries implemented various measures, such as pharmaceutical interventions, testing, vaccinations, and non-pharmaceutical interventions like lockdowns, school closures, and mask usage to seek to constrain the virus [2].

Sweden implemented a distinct approach towards the COVID-19 pandemic. Contrary to the strict lockdowns implemented by other countries, no legal restrictions were applied to the Swedish population [4]. The Swedish National Public Health Agency (Folkhälsomyndigheten) issued recommendations on behaviour to seek to reduce the infection rate, and the public was expected to follow these recommendations voluntarily [5]. While the policy managed to slow the economic downturn [6], Swedish healthcare was under immense pressure to cope with regular service and was overwhelmed with cases of COVID-19 [7]. Sweden’s unique strategy in tackling the COVID-19 pandemic has been reported to cause a more severe impact than in other developed countries, especially at the beginning of the pandemic [8]. As elaborated by Pashakhanlou, as of February 17, 2021, the death toll in Sweden was 1,241 per million inhabitants; in Norway, it was 111; in Finland, it was 130; and in Denmark, it was 399 per million inhabitants [8], [9]. IHME (The Institute for Health Metrics and Evaluation) COVID model estimated that many COVID-19 related deaths in Sweden were avoidable and in excess [10]. However, lower excess mortality from COVID-19 was reported for Sweden in 2022 compared to the other Nordic countries [11].

COVID-19 vaccination policies in European countries were similar, with the elderly population prioritized in the first phase of COVID-19 vaccinations [12]. Sweden also rolled out its COVID-19 vaccination program in several phases. Inhabitants in elderly care homes as well as staff and healthcare workers who work with risk groups were the targeted groups for the first phase, followed by individuals aged 70 or above, adults with functional impairments, and other medical healthcare professionals in phase two. The third phase targeted other adults in risk groups, while the rest of the population above 18 years old were included in the fourth phase [13].

The Swedish phases followed the principle of priority, where the group with the greatest need was the first to receive the vaccination. The definition of greatest need followed the WHO roadmap to assess the priority based on the risk of death and severe disease, vaccine effectiveness, and community acceptance [14]. Swedish National Public Health Agency was the official agency tasked with disseminating vaccinations to the Swedish population [13].

The scarcity of resources is the reason for performing health economic evaluations to aid decision-making in health care [15]. Economic evaluations provide a detailed account of the population health benefits and cost implications of healthcare intervention programs and form a basis for prioritising among alternative uses of healthcare resources [16]. The need for prioritisation was evident in the pandemic situation with the limited supply of COVID-19 vaccines.

One early Swedish study reported that the overall vaccination program against COVID-19 provided a value of €744–€956 per dose in the Swedish general population [17]. Early economic evidence reviews by Fu et al. reported both age-specific and general population cost-effectiveness analyses to inform global action about vaccination policies [18]. Another later systematic review reported that COVID-19 vaccination programs can be considered cost-effective, with the important factors: vaccine efficacy, dosage, and target population [19]. However, to our knowledge there was no study at the time this systematic review was composed that provides information on the cost-effectiveness of the age-based prioritization plan for COVID-19 vaccination in Sweden.

This systematic review thus aims at collecting evidence of the cost-effectiveness of COVID-19 vaccination in the elderly age-group, the adult age-group, and the children age-group from countries similar to Sweden and use this information to answer the research question: Can Sweden’s policy to prioritize vaccination of the elderly age-group before the age-groups adults and children, be considered cost-effective?

## Methods

This systematic review compares the cost-effectiveness of COVID-19 vaccination in the elderly, the adult, and the children age-groups. The PICOS (Population, Intervention, Control, Outcome and Study design) convention guided the search strategy. Two team members (TU and RP) carried out the study procedures independently. Any unresolved conflicts were addressed through consensus discussions facilitated by a third researcher (PJ), ensuring the rigor and reliability of the research process.

### Eligibility criteria

#### Study design

The inclusion criterion for the studies was economic evaluations relevant to COVID-19 vaccination in different age-groups. The types of economic evaluation included were cost-effectiveness analysis (CEA), cost-utility analysis (CUA), and cost-benefit analysis (CBA), with an explicit comparison between age-groups. Due to resource limitations, only reports in the English language were included.

#### Population

The population was specified as COVID-19 vaccinated European residents with age-group separation. Due to various definitions of age-groups in different countries, this study did not determine exact age limitations beforehand, but instead used age-groups as defined in the included studies.

Only studies performed in Europe were included, as these countries are similar to Sweden in demographics and associated COVID-19 disease risks, healthcare organization, and healthcare functionality (transferability purpose).

The geographical definition of Europe is the area from the Atlantic to the Ural Mountains, and from the Arctic to the Mediterranean [20].

#### Intervention

The intervention was COVID-19 vaccination, disregarding aspects such as the number of vaccine doses, boosters, type of vaccines, manufacturers, and period of vaccine administration.

#### Control

The control group was a population unvaccinated with COVID-19 vaccines.

#### Outcome

The included outcomes were incremental costs, cost per avoided infection, cost per QALY (quality-adjusted life-year), and net monetary benefit.

### Search strategy

#### Literature searches

The search strategy followed the PICOS framework, including studies published from January 2019 to February 2023. We searched four databases: MEDLINE, EMBASE, PsycInfo, and CINAHL using search strings adapted from the Sweden Health Technology Assessment Agency (SBU) and other relevant publications [21], [22]. Additional details are available in Appendix 1 and in the study repository at: https://doi.org/10.5878/hzf7-3485.

#### Complementary searches

Hand-search was performed on the reference list from the included reports to detect overlooked literature. The process was repeated until the reference lists were exhaustive of potential literature.

Grey literature searches were performed in selected websites: WHO (https://www.who.int/, The Swedish National Public Health Agency (https://www.folkhalsomyndigheten.se/) and EMA European Medicines Agency | (https://www.ema.europa.eu/en) with the same PICOS criteria and the same search terms.

Searches were also done in specialized health economic and HTA databases, including the Tuft CEA (Cost-Effectiveness Analysis) registry (tuftsmedicalcenter.org), Cochrane Library (https://www.cochranelibrary.com/), and INAHTA (The International Network of Agencies for Health Technology Assessment) HTA Database (inahta.org). The well-known NHS EED and HEED databases were excluded since they were no longer available [23].

### Title and abstract screening

The database search result was imported into the EndNote reference management system, and duplicate records deleted. The remaining records were then exported to Rayyan. Rayyan is an application to help expediting the screening of titles and abstracts [24]. The title and abstracts of the reports that did not fulfil eligibility criteria were excluded, while ambiguous reports were included into the full-text screening phase.

### Full-text screening

The full-text screening was documented in separate Microsoft Excel sheets filled out individually by each team member. The validation of these final lists was accomplished through consensus discussions. The reasons for excluding each report were documented (Appendix 2). The reporting of the result followed the guidelines from the Preferred Reporting Items for Systematic Review and Meta-analysis Protocols (PRISMA 2020) [25].

### Risk of bias assessment

The assessment of study quality and transferability to Sweden (risk of bias) was performed with the checklists of the Swedish HTA Agency SBU [26].

There are two versions of the SBU checklists: for assessment of model-based or trial-based economic evaluations. This study used the model-based checklist, to fit the included studies. The SBU checklists have been constructed to assist in identifying risk of bias as well as transferability to Swedish circumstances.

### Data extraction [55]

The extraction of data from included studies was based on the template by Mastrigt et al. [23], which is specific to health economics studies. The full data extraction table can be found in Appendix 4, while the summarized extraction table was based on the Swedish HTA Agency SBU template.

## Results

### Study selection

The database searches resulted in 6,053 records. After de-duplication, 5,720 reports remained. After the screening of title and abstract, 162 reports remained. The second stage of screening, where the full-text of each report was assessed for eligibility, reduced the number of reports to 3 reports, all reporting data on age-groups elderly and adult [27], [28], [29]. No reports with economic evidence for COVID-19 vaccination in the children age-group could be found. The reasons for exclusion of studies are reported in Appendix 2.

Extra hand-searches into the reference list of the three included studies resulted in one report [30]. Further screening of the reference list for the newly found report resulted in no relevant report. Grey literature searches in the websites WHO, The Swedish National Public Health Agency and EMA resulted in zero reports.

**Fig. 1:**
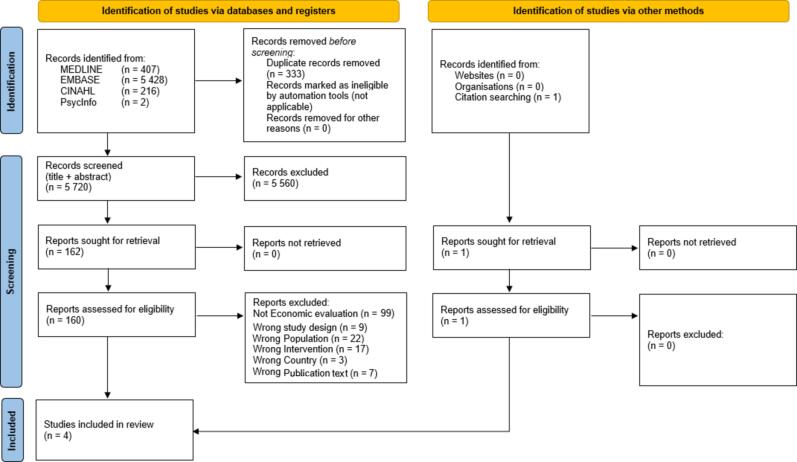
Results of included search: PRISMA Flowchart (2020).

Complementary searches into the three health economics databases resulted in no additional reports (see Fig. 1).

### Risk of bias assessment

The risk of bias assessment was performed on the four reports. Two reports were deemed to be of too low methodological quality and were excluded. One excluded study was considered low quality because there was a lack of information on the methods used and no sources for cost and effect were reported [29]. Another exclusion was due to inadequate information regarding the methods used and inadequate reporting of the calculations in the economic evaluation [30].

The other two reports [27], [28] were assessed as having a moderate risk of bias and were thus included in the review results. The risk of bias assessment checklist results for the four studies can be found in Appendix 3.

### Data extraction

#### Analytical approach and time horizon of the studies

Both reports' analytical approaches were model-based (see Table 1). Orlewska used a Markov model with the transition probabilities and disease progression from the Pfizer vaccine trial [31], combined with cost data from the country’s statistical agency. Orlewska’s time horizon was one year.

**Table 1:** Data extraction table.

Author Year Reference Country	Study design Population Setting Perspective	Intervention Control	Incremental cost	Incremental effect	ICER	Study quality and transferability
Orlewska 2022 [27] Poland	Model based (Markov) cost-effectiveness analysis with QALYs as effect measures Time period: costs 1 year, effects lifelong General population and 5 age groups: 30–39, 40–49, 60–69, >80 Healthcare perspective	I: Vaccination with Pfizer Comirnaty vaccine C: No vaccination	Costs reported in PLN (Polish Zloty) year 2020 No discounting Vaccine price: 38.05 PLN Administration: 61.24 PLN Ambulatory care: 370 PLN Hospitalization: 1026 PLN Hospitalization with ventilator: 4321 PLN	Discount rate 3.5 % QALY lost per death: General population 7.54 age groups: 30–39 19.9 40–49 17.1 60–69 10.4 >80 2.1	Cost (PLN) per QALY General population: 6,249 age groups: 30–39 67,823 40–49 28,135 60–69 cost saving >80 cost saving ICER of general population and age 30–39 is most affected by vaccine effectiveness, vaccine price, and SARS-CoV-2 infection rates. Vaccination of ages 60–69 and > 80, is cost-saving in most scenarios, and cost-effective (i.e. below the threshold of 3 × GDP/per capita of 147,024 PLN) for general population and all other age groups.	Moderate-high quality Moderate transferability to Sweden
Debrabant 2021 [28] Denmark	Model based (dynamic transmission model) cost-effectiveness analysis with QALYs as effect measures Time period: costs 6 months, effects > 35 years Age groups 18–60 and >60 years Healthcare perspective	I: Vaccination with assumed 100 % efficacy 4 scenarios: 1. Vaccination of 25 % of aged > 60 years 2. Vaccination of 25 % of aged 18–60 years 3. Vaccination of 15 % of aged 18–60 years and 25 % of aged > 60 years 4. Vaccination of 40 % of aged 18–60 years C: No vaccinated general population	Costs reported in DKK (Danish krona) year 2020 No discounting Vaccine price and administration cost in 3 scenarios: 300, 400, 500 DKK Hospitalization non-ICU: 16,495 DKK ICU patients aged 18–59: 26,028 DKK ICU patients aged > 60: 37,050 DKK Hospitalization with ventilator: 255,171 DKK Covid tests: 200 DKK Follow Up by GPs:146.25 DKK	Discount rate 4 % Total QALYs: 1. Q50^a^ 4490 2. Q50 1780 3. Q50 4850 4. Q50 2330	Cost (DKK) per QALY Scenarios 2 and 4 dominated (more costly and less effective than another scenario) Vaccine prices 300–500 DKK: Scenario 1 53,000–118,000 Scenario 3 319,000–803,000 Cost of hospitalization does not affect ICER. Mortality rate, vaccine efficacy, vaccine price, and inclusion of productivity costs affect ICER.	Moderate quality High transferability to Sweden

Abbreviations: I = intervention, C = control, PLN = Polish Zloty, DKK = Danish krona, QALY = Quality-adjusted life-years, GDP = Gross Domestic Product, ICER = incremental cost-effectiveness ratio,

^a^Q50 is the weighted median.

Debrabant used the dynamic transition model developed by the Danish Ministry of Health. The model is reported to be the most suitable for communicable diseases and vaccination programs [32]. The time horizon for Debrabant was six months.

#### Study perspective and country setting

The perspective recommended for Swedish health economic evaluations is the societal perspective [33]. The perspective includes direct healthcare costs, productivity costs and costs to the patient and family [33]. Debrabant's and Orlewska's studies focused on the healthcare perspective, while Debrabant included productivity costs as a sensitivity analysis. The method for measuring productivity costs described in the Debrabant study was similar to the human capital method in the Swedish HTA agency method handbook. There is a concern that this method overestimates the productivity costs [33].

#### Population, intervention, and control

In Orlewska's study, the population was composed of 5 groups: the general population and four age-groups (30–39 years, 40–49 years, 60–69 years, and +80 years). The same age composition was applied to the control group.

Debrabant divided the intervention groups into four scenarios based on two age-groups (18–60 years and +60 years). Contrary to Orlewska, the control group in Debrabant did not replicate the intervention composition; instead, Debrabant compared the intervention with only one group, the non-vaccinated Danish general population.

Orlewska's intervention was the Comirnaty vaccine (Pfizer BioNTech), the vaccine given in Poland's COVID-19 vaccination program. Debrabant did not state the vaccine name or the producer in the study. However, Debrabant reported that the vaccine’s effectiveness in the study was 95 percent, which is similar to the effectiveness of the Pfizer BioNTech vaccine [31].

#### Resource identification, quantification, and valuation

Both reports reported the resource value in local currencies and considered the vaccine's price and administration cost. Orlewska quoted the exact value for the vaccine's price and administration cost, while Debrabant provided a range of values combining the vaccine's price and administration cost. Debrabant utilized three scenarios of vaccine costs (300 DKK, 400 DKK, and 500 DKK, equivalent to €40.23, €53.64, and €67.05). Both reports also calculated hospitalization costs. Debrabant included the cost of COVID-19 tests and after-test follow-up by physicians in the total resources used, while Orlewska limited the identification of the resource use to hospitalization.

To assess transferability to Sweden, the cost of hospitalization and other relevant treatments, as described in Debrabant and Orlewska, are compared with the cost in Sweden. The cost for hospitalization, vaccine administration, and after-test follow-up by physicians in Sweden is taken from the official register from Sveriges Kommuner och Regioner (SKR) website [34] and the pricelist from the southern region of Sweden (Region Skåne) [35].

As only the daily price is provided in the Swedish price list, the total is calculated by multiplying the price per day with the length of stay of hospitalization and ICU (Intensive Care Unit) following the base case cost data from Orlewska. The cost for the COVID-19 test [36] and the vaccine price [37] were estimated from unofficial reports in newsletters.

The healthcare cost in these three countries was different, especially for vaccine administration per dosage (Table 2). Regarding ICU cost, Denmark and Sweden were more expensive than Poland.

**Table 2:** Cost comparison between Denmark, Sweden, and Poland, in 2020 (in €).

Parameters	Denmark (in €)	Sweden (in €)	Poland (in €)
Hospitalisation (without ICU)	2212	3802	2664
Patients aged 18–59 years (ICU)	3491	4620	2664
Patients aged +60 years (ICU)	4969	4620	2664
Hospitalisation in respirator	34,226	29,986	14,155
Tests	27	19	N/A
After test follow up	20	175	N/A
Vaccine Administration/dose	20	102	14
Vaccine price/dose	15	15	9

N/A = not available.

#### Effect identification, measurement, and valuation

While both reports reported QALYs (quality-adjusted life-years) to measure the effectiveness of the COVID-19 vaccination program, Debrabant also utilized LY (life-years).

Debrabant and Orlewska utilized QALY measurement from other studies [27], [28]. In Sweden, at the time this systematic review was composed, the existing reports regarding COVID-19-related QALYs in Sweden were provided by The Swedish Institute for Health Economics [38] and Persson et al. [39]. The transferability assessment of outcome results (QALY) from Debrabant’s and Orlewska's studies was made against these two existing reports about Sweden.

The excess deaths in Sweden due to COVID-19 in the first six months of 2020 was 5,310 deaths, translated to a loss of 32,082 QALYs [38]. This equals 6.04 QALY lost/death. The report by Persson et al. suggested a similar number (6.07 QALY lost/death) [39].

Debrabant reported lost QALYs of 5,410 for the non-vaccinated general population in Denmark (control group) for six months of 2020, compared to the 32,000 lost QALYs in Sweden during the same time period. The voluntary COVID-19 policy and the larger population in Sweden (Sweden's population was twice of Denmark’s) could be the factors that contributed to the higher number of lost QALYs in Sweden (six times higher than in Denmark).

**Fig. 2:**
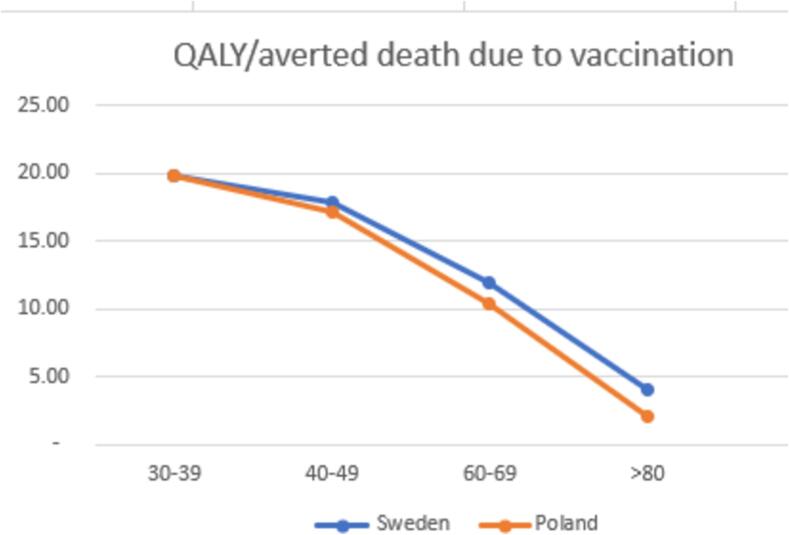
The differences in the effect between Poland and Sweden.

Orlewska provided information on the excess expected number of deaths per age-group among non-vaccinated compared to vaccinated. Persson et al. provided only the numbers among non-vaccinated [39]. By combining information from Orlewska and Persson, the QALYs gained and the number of averted deaths due to vaccination can be estimated as provided in Fig. 2 (detailed calculation in Appendix 5).

The estimates for Sweden were similar to the Orlewska study for age-groups 30–39, 40–49, and 60–69. However, there were slight differences for the age-group 80 and older; Orlewska's calculation resulted in 2.12 QALYs, while the estimates for Sweden are 4.06 QALYs. This implies that vaccination of the elderly is more cost-effective in Sweden than in Poland, as the gains in health for the Swedish elderly would be higher.

#### ICER

Debrabant used no specific willingness-to-pay threshold cost per QALY in its assessment of the cost-effectiveness of the Danish COVID-19 vaccination program. Instead, Debrabant compared the results with other public health interventions. For the transferability assessment, we apply the often used 500,000 SEK (€ 43,000)/QALY threshold in Sweden [40] to the Debrabant result [28]. This indicates that the Debrabant scenario 1, i.e. vaccinating 25 % of the population aged 60 and older, would have been cost-effective in Sweden, i.e., below the Swedish willingness-to-pay threshold.

If productivity costs are to be considered, the decision to vaccinate 15 % of the Swedish population aged 18–59 and 25 % of the Swedish population aged 60 and older (scenario 3 of Debrabant’s study) would have resulted in a higher ICER, ranging from 500,000 to 1,000,000 SEK (€ 43,000–86,000)/QALY). This is higher than the willingness-to-pay threshold of Sweden. Omitting productivity costs, the decision to vaccinate the same groups would have resulted in a very high cost per QALY (>1,000,000 SEK (€ 86,000)/QALY).

Orlewska compared the program’s cost-effectiveness to the willingness-to-pay threshold generally used in Poland: three times GDP or 147,024 PLN (€ 31,904)/QALY in 2020. The ICER reported for the age-group 30–39 is 67,823 PLN (€ 14,718)/QALY (cost-effective), for the age-group 40–49 28,135 PLN (€ 6,105)/QALY (cost-effective), and for ages 60 years and older the vaccination is cost saving.

The highest ICER in Orlewska was for ages 30–39 at 67,823 PLN (€ 14,718), equal to around 160,000 SEK (€13,760), which would be considered cost-effective in Sweden. This aligns with the conclusion of the Orlewska study that vaccinating any age-group was cost-effective.

#### Sensitivity analysis

In the study by Orlewska, a deterministic sensitivity analysis was conducted, considering both best- and worst-case scenarios for each parameter. The parameters analysed included virus attack rate, case fatality rate, number of hospitalized patients, vaccine efficacy, treatment cost, length of hospitalization, vaccination cost, and combinations of these factors. The analysis revealed that the ICER was particularly sensitive to vaccine effectiveness, price, and infection rate, but only within the 30–39 age group.

In contrast, Debrabant did not specify the type of sensitivity analysis used but focused on parameters such as vaccination coverage (70 % of the population), mortality rate, QALY parameters, vaccine efficacy, and hospitalization costs. Notably, Debrabant included productivity costs in the sensitivity analysis. Debrabant calculated productivity costs by multiplying the number of days of sick leave due to COVID-19 disease with earnings per hour, employment rate, and the number of working hours per day. The findings indicated that ICER was sensitive to mortality rate and vaccine efficacy, while the overall cost-effectiveness profile of the program was strongly influenced by vaccine price and the inclusion of productivity costs in the cost calculations.

## Discussion

This systematic review sought to investigate whether the COVID-19 vaccination policy of Sweden, similar to the policy in many other countries, constituted a wise use of the resources available. Vaccination of the elderly population was prioritized, before the other age-groups adults and children. Two studies were included, one each from Poland and Denmark, and the review could conclude that in those two countries, vaccinating the age-group 60 years and older was cost-effective compared to vaccinating other age-groups.

However, the systematic review found that those findings were not transferable to the Swedish setting, mainly due to major differences in cost and effect data between Sweden and the countries Denmark and Poland. No relevant study could be found that compared the cost-effectiveness of vaccinating the children in comparison with other age-groups. The research question of whether Sweden’s strategy to vaccinate the elderly population before the other age-groups was the cost-effective option could thus not be answered by this review.

Our results indicate that vaccine price is the driving factor in determining whether the COVID-19 vaccination program was cost-effective or not. It was found that if the vaccination price in Denmark was 300 DKK or less, then the covid-19 vaccination program could be cost-effective for the adult population as well. Similarly, incorporating productivity costs in the sensitivity analysis calculation could shift the balance of COVID-19 vaccination for an adult group to becoming cost-effective, with the pre-condition of a low vaccine price. Fu et al and Chang et al. similarly concluded that vaccine price determines cost effectiveness of Covid-19 vaccination program [18], [19].

Strengths of this study included the unbiased selection process with independent team members, guidance from an experienced health economist, adherence to systematic review guidelines, and a strict risk of bias assessment that involved both methodological study quality and transferability to the selected setting. The study also included a comprehensive hand-search of reference lists.

The study was limited by only including two reports, potentially due to the specificity of PICOS criteria to European countries and the relatively short period between the pandemic onset and the review. This contrasts with the review by Fu et al. [18] which included reports from all countries. However, it is worth mentioning that the search strategy conducted for this study captured all the reports in Fu et al. (Appendix 2).

The study conducted by Fu et al. also discussed the benefits of booster doses as it was found that booster doses reduce the infection rate, hospitalization rate, and mortality. Another limitation of our study is that we only investigated the COVID-19 vaccination based on age-groups, and we overlooked the consideration of prioritization based on any previous SARS-CoV-2 infection. Booster vaccinations and vaccinations following infections would lead to a different cost-effectiveness profile than the initial vaccination considered in this review.

This study highlighted the need for more evidence on the effectiveness and cost-effectiveness of vaccinations for the children's age-group. Some publications emphasized the potential benefits of vaccinating children and adolescents’ groups, including reducing hospitalizations, deaths, and long-term effects of COVID-19 [41], [42].

For example, vaccinating the unvaccinated children population is reported to reduce the risk of hospitalization by 93 % [43]. Moreover, the US CDC has recommended vaccinating the children and adolescent population as early as possible [43]. Similarly, for the UK, a study estimated that vaccinating the children population can reduce COVID-19-related hospitalization, mortality, and long COVID cases by 21 %, 18 %, and 27 %, respectively [44].

According to Lang et al., an empty systematic review is a “systematic literature review that does not identify any eligible studies for inclusion in the analysis” [45]. Gray argued that empty systematic literature reviews could help identify the potential research gaps when there is a lack of evidence [46]. An empty systematic literature review can occur for several reasons, one of which may be the specificity of the research question. In our case, we believe the main explanation for the lack of cost-effectiveness studies on the children population is because COVID-19 is a relatively new virus, and there are still uncertainties about how the vaccine might affect the children population [47].

The decline in mortality rate and disease severity between the first and second phases of COVID-19 along with the increased vaccination production mean that vaccine shortage was an issue mainly in the first phase of COVID-19. Yet, to prepare for future epidemics, the prioritization of vaccine allocation based on biological consideration (age) should be studied, to analyse whether this prioritization strategy was cost-effective or not. The high costs associated with vaccinations of full populations coupled with the scarce resources of healthcare systems make economic evaluations of vaccination programs crucial [48].

Economic evaluation provides robust evidence-based decision-making by providing guidelines and recommendations to policymakers by providing a comprehensive analysis of both cost and benefits; see for example the WHO recommendations [49].

The costs associated with the vaccine administration are often divided into two parts, i.e., direct costs and productivity costs (also called indirect costs). The direct costs include the vaccine purchasing prices, distribution of the vaccines, and the number of doses required. Cost savings arise from the cases of disease averted, such as decreases in hospitalisations, in acute care, and in healthcare staff costs. Productivity costs (indirect costs) constitute losses of working capacity both due to the vaccination in terms of patient time for the healthcare visit and due to possible negative side effects, such as the reported rates of sickness following the initial doses [50].

These components of productivity costs are considered a cost for the wider economy and included in a societal perspective. The benefits of vaccination programs are the population health gains, that could be measured as mortality and morbidity. The recommended health measure among health economists is however QALYs (quality-adjusted life-years) that combine life-years lost with health-related quality-of life [51].

It is also recommended to choose the right health economic modelling techniques for estimating the consequences of vaccination programs [52]. The most important model is the Disease Transmission Model, which allows the decision-makers to calculate disease transmission and highlight the herd immunity effects. Another common model is the Markov model, that is used for estimating long-term outcomes [52]. A full account of the benefits and costs connected to vaccination programs, as in the studies with high to moderate study quality included in this review, enable decision-makers to consider the cost-effectiveness of the programs when prioritizing between alternative uses of health care resources [50].

### The implication to health policy and practice/clinical

In Sweden, three ethical principles guide priority setting in the health service [53]. In some countries, along with Sweden, the early COVID-19 vaccination strategy was based only on two of these principles: the human dignity principle and the needs and solidarity principle. The third ethical principal, the reasonable balance between costs and patient benefits, was not explicitly considered.

Early in the epidemic, it became clear from studies conducted both within and outside Sweden that COVID-19 vaccination is cost-effective at the general population level [17], [18]. This systematic review contributes to confirming that the COVID-19 vaccination policy in the European continent, which prioritizes the elderly population, is valid based on cost-effectiveness grounds. This is important to study since the initial considerations of the policy were not based on concrete economic evidence.

As COVID-19 continues to evolve, comprehending its impact on children and the implications of vaccinating them gains significance. While severe cases are less common in children [54], generating more evidence on the health effects of COVID-19 among children, as well as the safety and effectiveness of COVID-19 vaccines in this population and performing economic evaluations of vaccination among children will be important for guiding public health policies and resource allocation to ensure optimal health outcomes for all.

## Conclusion

This systematic review concludes that prioritizing vaccination of the elderly population (60 years and older) is considered cost-effective in comparison with vaccinating the adult population (18–59 years). The included studies, one each from Denmark and Poland, are however not fully transferable to the Swedish context. Nevertheless, the available cost-effectiveness evidence indicates that the choice made by many countries in the resource constrained situation at the beginning of the COVID-19 vaccination programs was sound and justified also on cost-effectiveness grounds.

This manuscript is a combined work of two master students’ theses that are published individually in the University of Gothenburg library database: https://hdl.handle.net/2077/77409 and https://hdl.handle.net/2077/77472.

All authors attest that they meet the ICMJE criteria for authorship.

## Funding source

This research did not receive any specific grant from funding agencies in the public, commercial, or not-for-profit sectors.

## CRediT authorship contribution statement

**T. Untung:** Writing – review & editing, Writing – original draft, Methodology. **R. Pandey:** Writing – review & editing, Writing – original draft. **P. Johansson:** Writing – review & editing, Supervision.

## Declaration of competing interest

The authors declare that they have no known competing financial interests or personal relationships that could have appeared to influence the work reported in this paper.

## Data Availability

Data presented in this article are available from the online repository DORIS Swedish National Data under the title, ‘‘The cost-effectiveness of Covid-19 vaccination in age groups children, adults and elderly in Europe – A systematic review” with digital object identifier https://snd.se/sv/catalogue/dataset/2023-168/1.
