# Supplementary material for: The cost-effectiveness of COVID-19 vaccination program among age-groups children, adults, and elderly in Europe: A systematic review
Source: Vaccine X. 2024 Nov 8;21:100580. doi: 10.1016/j.jvacx.2024.100580 (PMC11615606; doi:10.1016/j.jvacx.2024.100580)
Supplement: Supplementary Data 1 [file mmc1.pdf]

Appendix 1.

Embase via GU library website (09 February 2023)

Title: The economic evaluation and cost benefit analysis for COVID-19 vaccination

Search terms		Items found
Population: COVID-19[1)]		
1	‘coronavirus disease 2019’/de	289,118
2	(COVID* or sars cov* or sarscov* or corona virus or coronavirus or ncov):ti,ab,kw	148,431
3	1 or 2	336,775
Intervention: Vaccination		
4	Vaccine	513,857
5	Vaccination	313,661
6	Vaccin	2,159
7	Immunization	217,680
8	4 or 5 or 6 or 7	689,691
Health economic aspects /Economic aspects[2)]		
9	('health economics'/de OR 'economic evaluation'/exp OR 'health care cost'/exp OR 'pharmacoeconomics'/exp OR econom*:ab,ti OR cost:ab,ti OR costs:ab,ti OR costly:ab,ti OR costing:ab,ti OR price:ab,ti OR prices:ab,ti OR pricing:ab,ti OR pharmacoeconomic*:ab,ti OR (expenditure*:ti,ab NOT energy:ti,ab) OR ((value NEXT/2 money):ab,ti) OR budget*:ab,ti) NOT (((metabolic NEXT/2 cost):ab,ti) OR (((energy OR oxygen) NEXT/2 cost):ab,ti) OR (((energy OR oxygen) NEAR/2 expenditure):ab,ti))	1,741,771
Combined sets		
10	3 and 8 and 9	5,094
Health economic aspects /Economic aspects[2)]		
11	'economics'/mj	28,749
12	'cost'/exp/mj	90,454
13	economic NEAR/2 model*	9,696
14	'cost minimi*':ti,ab,kw OR 'cost utilit*':ti,ab,kw OR 'health utilit*':ti,ab,kw OR 'economic evaluation*':ti,ab,kw OR 'economic review*':ti,ab,kw OR 'cost outcome':ti,ab,kw OR 'cost analys$s':ti,ab,kw OR 'economic analys$s':ti,ab,kw OR 'budget* impact analys$s':ti,ab,kw	62,139
15	'cost effective*':ti,kw OR pharmacoeconomic*:ti,kw OR 'pharmaco economic*':ti,kw OR 'cost benefit':ti,kw OR costs:ti,kw	126,148
16	'life year':ab,kw OR 'life years':ab,kw OR qaly*:ab,kw OR 'cost-benefit analys$s':ab,kw OR 'cost-effectiveness analys$s':ab,kw	58,592

17	(cost:ti,kw OR economic*:ti,kw) AND (costs:ab OR 'cost effectiveness':ab OR markov:ab)	109,515
18	11 or 12 or 13 or 14 or 15 or 16 or 17	301,500
Combined sets		
19	3 and 8 and 18	329
Health economic aspects /Economic aspects[2)]		
20	(cost$ NEAR/2 (illness OR disease OR sickness)):ti,ab	7,497
21	(burden$ NEAR/2 (illness OR disease$ OR condition$ OR economic*)):ti,ab	81,378
22	'quality-adjusted life years':ti,ab OR 'quality adjusted life years':ti,ab OR qaly$:ti,ab	28,422
23	'quality adjusted life year'/de	33,538
24	'cost of illness'/de	20,887
25	'health care cost'/exp	332,392
26	('out of pocket' NEAR/2 (payment$ OR expenditure$ OR cost$ OR spending OR expense$)):ti,ab	9,175
27	(expenditure$ NEAR/3 (health OR direct OR indirect)):ti,ab	13,798
28	((adjusted OR 'quality adjusted') NEAR/2 year$):ti,ab	39,741
29	20 or 21 or 22 or 23 or 24 or 25 or 26 or 27 or 28	458,639
Combined sets		
30	3 and 8 and 29	1,414
31	10 or 19 or 30	5,425

1) COVID-19 and pregnancy (sbu.se)
2) Bilaga 1 Sökdokumentation (sbu.se)

/de= Term from the EMTREE controlled vocabulary

/exp= Includes terms found below this term in the EMTREE hierarchy

/mj = Major Topic

:ab = Abstract

:au = Author

:ti = Article Title

:ti:ab = Title or abstract

* = Truncation

“ “ = Citation Marks; searches for an exact phrase

NEAR/x = Requests terms that are within 'x' words of each other in either direction

COCHRANE

Cochrane via Wiley (22 March 2023)

Title: The economic evaluation and cost benefit analysis for COVID-19 vaccination

Search terms		Items found
Population: COVID-19[1)]		
1	Exp Coronavirus/	9,026
2	Exp Coronavirus Infections/	566
3	(COVID* or sars cov* or sarscov* or corona virus or coronavirus or ncov).ab,kf,ti	15,874
4	1 or 2 or 3	15,916
Intervention: Vaccination		
5	Vaccine	26,104
6	Vaccination	17,950
7	Vaccin	538
8	Immunization	9,682
9	5 or 6 or 7 or 8	31,396
Combined sets		
10	4 and 9	2,272
Health economic aspects /Economic aspects[2)]		
11	MeSH descriptor: [Economics] this term only	51
12	MeSH descriptor: [Costs and Cost Analysis] explode all trees	14,254
13	MeSH descriptor: [Economics, Dental] this term only	2
14	MeSH descriptor: [Economics, Hospital] explode all trees	824
15	MeSH descriptor: [Economics, Medical] this term only	32
16	MeSH descriptor: [Economics, Nursing] this term only	13
17	MeSH descriptor: [Economics, Pharmaceutical] this term only	121
18	(((economic* OR cost OR costs OR costly OR costing OR price OR prices OR pricing OR pharmacoeconomic* OR (expenditure* NOT energy) OR (value NEAR/2 money) OR budget*) NOT (((energy OR oxygen) NEAR/10 cost) OR (metabolic NEAR/10 cost) OR ((energy OR oxygen) NEAR/10 expenditure)))):ti,ab,kw	96,915
19	11-18	97,008
Combined sets		
20	10 and 19	118
Health economic aspects /Economic aspects[2)]		
21	([mh ^Economics[mj]])	0
22	([mh "costs and cost analysis"[mj]])	855
23	(economic NEAR/2 model*)	931

24	(((cost next/1 minimi*) OR cost-utilit* OR (health next/1 utilit*) OR (economic next/1 evaluation*) OR (economic next/1 review*) OR "cost outcome" OR "cost analysis" OR "cost analyses" OR "economic analysis" OR "economic analyses" OR (budget* next/1 impact next/1 analysis) OR (budget* next/1 impact next/1 analyses))):ti,ab,kw	12,014
25	((cost-effective* OR pharmacoeconomic* OR pharmaco-economic* OR cost-benefit OR costs)):ti,ab,kw	58,128
26	(("life year" OR "life years" OR qaly*)):ti,ab,kw	7,314
27	(("cost-benefit analysis" OR "cost-benefit analyses" OR "cost-effectiveness analysis" OR "cost-effectiveness analyses")):ti,ab,kw	20,872
28	((cost OR economic*)):ti,ab,kw AND ((costs OR cost-effectiveness OR markov)):ab	32,679
29	21-28	61,425
Combined sets		
30	10 and 29	42
Health economic aspects /Economic aspects[2)]		
31	(((cost OR costs) NEAR/2 (illness OR disease OR sickness))):ti,ab,kw	1,947
32	(((burden OR burdens) NEAR/2 (illness OR disease OR diseases OR condition OR conditions OR economic*))):ti,ab,kw	4,726
33	(("quality-adjusted life years" OR "quality adjusted life years" OR QALY OR QALYs)):ti,ab,kw	5,944
34	MeSH descriptor: [Quality-Adjusted Life Years] this term only	1,930
35	MeSH descriptor: [Cost of Illness] this term only	1,054
36	MeSH descriptor: [Health Expenditures] this term only	332
37	((out-of-pocket NEAR/2 (payment OR payments OR expenditure OR expenditures OR cost OR costs OR spending OR expense OR expenses))):ti,ab,kw	90,694
38	(((expenditure OR expenditures) NEAR/3 (health OR direct OR indirect))):ti,ab,kw	1,072
39	(((adjusted OR quality-adjusted) NEAR/2 (year OR years))):ti,ab,kw	7,089
40	31-39	95,170
Combined sets		
41	10 and 40	107
42	20 or 30 or 41	118

1) COVID-19 and pregnancy (sbu.se)
2) Bilaga 1 Sökdokumentation (sbu.se)

The search result, usually found at the end of the documentation, forms the list of abstracts.

:au = Author

MeSH = Term from the Medline controlled vocabulary, including terms found below this term in the MeSH hierarchy

this term only = Does not include terms found below this term in the MeSH hierarchy

:ti = title

:ab = abstract

:kw = keyword

* = Truncation

“ “ = Citation Marks; searches for an exact phrase

NEAR/x = Finds the terms when they are within x words of each other. Terms can appear in either order

NEXT/x = Finds the terms when they appear next to each other. Terms must appear in the order specified

CDSR = Cochrane Database of Systematic Review

CENTRAL = Cochrane Central Register of Controlled Trials, “trials”

CINAHL

Thursday, February 09, 2023 6:28:26 PM

#	Query	Limiters/Expanders	Last Run Via	Results
S1	TI "severe acute respiratory syndrome coronavirus 2" OR AB "severe acute respiratory syndrome coronavirus 2"	Expanders - Apply equivalent subjects<br>Search modes - Find all my search terms	Interface - EBSCOhost Research Databases<br>Search Screen - Advanced Search<br>Database - CINAHL with Full Text	553
S2	TI "2019 nCoV" OR AB "2019 nCoV"	Expanders - Apply equivalent subjects<br>Search modes - Find all my search terms	Interface - EBSCOhost Research Databases<br>Search Screen - Advanced Search<br>Database - CINAHL with Full Text	2
S3	TI 2019nCoV OR AB 2019nCoV	Expanders - Apply equivalent subjects<br>Search modes - Find all my search terms	Interface - EBSCOhost Research Databases<br>Search Screen - Advanced Search<br>Database - CINAHL with Full Text	2,629
S4	TI "2019-nCov" OR AB "2019-nCov"	Expanders - Apply equivalent subjects<br>Search modes - Find all my search terms	Interface - EBSCOhost Research Databases<br>Search Screen - Advanced Search<br>Database - CINAHL with Full Text	71
S5	TI CoVID-19 OR AB CoVID-19	Expanders - Apply equivalent subjects<br>Search modes - Find all my search terms	Interface - EBSCOhost Research Databases<br>Search Screen - Advanced Search<br>Database - CINAHL with Full Text	21,841
S6	TI SARS-CoV-2 OR AB SARS-CoV-2	Expanders - Apply equivalent subjects<br>Search modes - Find all my search terms	Interface - EBSCOhost Research Databases<br>Search Screen - Advanced Search	2,629

Internal

			Database - CINAHL with Full Text	
S7	TI "SARS CoV 2" OR AB "SARS CoV 2"	Expanders - Apply equivalent subjects<br>Search modes - Find all my search terms	Interface - EBSCOhost Research Databases<br>Search Screen - Advanced Search<br>Database - CINAHL with Full Text	20
S8	TI CoVid OR AB CoVid	Expanders - Apply equivalent subjects<br>Search modes - Find all my search terms	Interface - EBSCOhost Research Databases<br>Search Screen - Advanced Search<br>Database - CINAHL with Full Text	21,841
S9	TI nCov OR AB nCov	Expanders - Apply equivalent subjects<br>Search modes - Find all my search terms	Interface - EBSCOhost Research Databases<br>Search Screen - Advanced Search<br>Database - CINAHL with Full Text	7
S10	TI "novel coronavirus" OR AB "novel coronavirus"	Expanders - Apply equivalent subjects<br>Search modes - Find all my search terms	Interface - EBSCOhost Research Databases<br>Search Screen - Advanced Search<br>Database - CINAHL with Full Text	521
S11	TI "new coronavirus" OR AB "new coronavirus"	Expanders - Apply equivalent subjects<br>Search modes - Find all my search terms	Interface - EBSCOhost Research Databases<br>Search Screen - Advanced Search<br>Database - CINAHL with Full Text	67
S12	TI "coronavirus 2019" OR AB coronavirus 2019"	Expanders - Apply equivalent subjects<br>Search modes - Find all my search terms	Interface - EBSCOhost Research Databases<br>Search Screen - Advanced Search<br>Database - CINAHL with Full Text	2,163
S13	TI "SARS coronavirus 2" OR AB "SARS	Expanders - Apply equivalent subjects	Interface - EBSCOhost Research Databases	3

	coronavirus 2"	Search modes - Find all my search terms	Search Screen - Advanced Search<br>Database - CINAHL with Full Text	
S14	TI CoVID19 OR AB CoVID19	Expanders - Apply equivalent subjects<br>Search modes - Find all my search terms	Interface - EBSCOhost Research Databases<br>Search Screen - Advanced Search<br>Database - CINAHL with Full Text	21,841
S15	S1 OR S2 OR S3 OR S4 OR S5 OR S6 OR S7 OR S8 OR S9 OR S10 OR S11 OR S12 OR S13 OR S14	Expanders - Apply equivalent subjects<br>Search modes - Find all my search terms	Interface - EBSCOhost Research Databases<br>Search Screen - Advanced Search<br>Database - CINAHL with Full Text	22,872
S16	MH "Severe Acute Respiratory Syndrome"	Expanders - Apply equivalent subjects<br>Search modes - Find all my search terms	Interface - EBSCOhost Research Databases<br>Search Screen - Advanced Search<br>Database - CINAHL with Full Text	433
S17	MH "SARS Virus"	Expanders - Apply equivalent subjects<br>Search modes - Find all my search terms	Interface - EBSCOhost Research Databases<br>Search Screen - Advanced Search<br>Database - CINAHL with Full Text	74
S18	TI "Severe acute respiratory syndrome" OR AB "Severe acute respiratory syndrome"	Expanders - Apply equivalent subjects<br>Search modes - Find all my search terms	Interface - EBSCOhost Research Databases<br>Search Screen - Advanced Search<br>Database - CINAHL with Full Text	953
S19	TI SARS OR AB SARS	Expanders - Apply equivalent subjects<br>Search modes - Find all my search terms	Interface - EBSCOhost Research Databases<br>Search Screen - Advanced Search<br>Database - CINAHL with Full Text	776

S20	TI SARS-CoV OR AB SARS-CoV	Expanders - Apply equivalent subjects<br>Search modes - Find all my search terms	Interface - EBSCOhost Research Databases<br>Search Screen - Advanced Search<br>Database - CINAHL with Full Text	86
S21	S16 OR S17 OR S18 OR S19 OR S20	Expanders - Apply equivalent subjects<br>Search modes - Find all my search terms	Interface - EBSCOhost Research Databases<br>Search Screen - Advanced Search<br>Database - CINAHL with Full Text	1,640
S22	S15 OR S21	Expanders - Apply equivalent subjects<br>Search modes - Find all my search terms	Interface - EBSCOhost Research Databases<br>Search Screen - Advanced Search<br>Database - CINAHL with Full Text	23,625
S23	"vaccination*"	Expanders - Apply equivalent subjects<br>Search modes - Find all my search terms	Interface - EBSCOhost Research Databases<br>Search Screen - Advanced Search<br>Database - CINAHL with Full Text	9,778
S24	“immunization*”	Expanders - Apply equivalent subjects<br>Search modes - Find all my search terms	Interface - EBSCOhost Research Databases<br>Search Screen - Advanced Search<br>Database - CINAHL with Full Text	9,970
S25	"immunisation*"	Expanders - Apply equivalent subjects<br>Search modes - Find all my search terms	Interface - EBSCOhost Research Databases<br>Search Screen - Advanced Search<br>Database - CINAHL with Full Text	4,263
S26	"immunize*"	Expanders - Apply equivalent subjects<br>Search modes - Find all my search terms	Interface - EBSCOhost Research Databases<br>Search Screen - Advanced Search	499

Internal

			Database - CINAHL with Full Text	
S27	"immunise*"	Expanders - Apply equivalent subjects<br>Search modes - Find all my search terms	Interface - EBSCOhost Research Databases<br>Search Screen - Advanced Search<br>Database - CINAHL with Full Text	57
S28	"vaccine*"	Expanders - Apply equivalent subjects<br>Search modes - Find all my search terms	Interface - EBSCOhost Research Databases<br>Search Screen - Advanced Search<br>Database - CINAHL with Full Text	16,431
S29	"shots*"	Expanders - Apply equivalent subjects<br>Search modes - Find all my search terms	Interface - EBSCOhost Research Databases<br>Search Screen - Advanced Search<br>Database - CINAHL with Full Text	530
S30	S23 OR S24 OR S25 OR S26 OR S27 OR S28 OR S29	Expanders - Apply equivalent subjects<br>Search modes - Find all my search terms	Interface - EBSCOhost Research Databases<br>Search Screen - Advanced Search<br>Database - CINAHL with Full Text	22,131
S31	((MH "Economics") OR (MH "Costs and Cost Analysis+") OR (MH "Health Care Costs+") OR (MH "Economics, Dental") OR (MH "Nursing Costs") OR (MH "Economics, Pharmaceutical") OR TI (econom* or cost or costs or costly or costing or price or prices or pricing or pharmacoeconomic*) OR AB (econom* or cost or costs or costly or costing or price or prices	Expanders - Apply equivalent subjects<br>Search modes - Find all my search terms	Interface - EBSCOhost Research Databases<br>Search Screen - Advanced Search<br>Database - CINAHL with Full Text	91,220

Internal

	or pricing or pharmacoeconomic*) OR TI (Expenditure* NOT energy) OR AB (Expenditure* NOT energy) OR TI (value N1 money) OR AB (value N1 money) OR TI (budget*) OR AB (budget*)) NOT (TI (Metabolic W2 cost) OR AB (Metabolic W2 cost) OR TI ((energy OR oxygen) W2 cost) OR AB ((energy OR oxygen) W2 cost) OR TI ((Energy OR oxygen) W2 expenditure) OR AB ((Energy OR oxygen) W2 expenditure))			
S32	S22 AND S30 AND S31	Expanders - Apply equivalent subjects<br>Search modes - Find all my search terms	Interface - EBSCOhost Research Databases<br>Search Screen - Advanced Search<br>Database - CINAHL with Full Text	199
S33	(MM "Economics")	Expanders - Apply equivalent subjects<br>Search modes - Find all my search terms	Interface - EBSCOhost Research Databases<br>Search Screen - Advanced Search<br>Database - CINAHL with Full Text	2,524
S34	(MM "Costs and Cost Analysis+")	Expanders - Apply equivalent subjects<br>Search modes - Find all my search terms	Interface - EBSCOhost Research Databases<br>Search Screen - Advanced Search<br>Database - CINAHL with Full Text	11,341
S35	TX (economic N1 model*)	Expanders - Apply equivalent subjects<br>Search modes - Find all my search terms	Interface - EBSCOhost Research Databases<br>Search Screen - Advanced Search	2,182

			Database - CINAHL with Full Text	
S36	TI ("cost minimi*" or cost-utilit* or "health utilit*" or "economic evaluation*" or "economic review*" or "cost outcome" or "cost analys#s" or "economic analys#s" or "budget* impact analys#s") OR AB ("cost minimi*" or cost-utilit* or "health utilit*" or "economic evaluation*" or "economic review*" or "cost outcome" or "cost analys#s" or "economic analys#s" or "budget* impact analys#s"	Expanders - Apply equivalent subjects<br>Search modes - Find all my search terms	Interface - EBSCOhost Research Databases<br>Search Screen - Advanced Search<br>Database - CINAHL with Full Text	5,992
S37	TI cost-effective* or pharmacoeconomic* or pharmaco-economic* or cost-benefit or costs	Expanders - Apply equivalent subjects<br>Search modes - Find all my search terms	Interface - EBSCOhost Research Databases<br>Search Screen - Advanced Search<br>Database - CINAHL with Full Text	59,817
S38	AB "life year" or "life years" or qaly* or "cost-benefit analys#s" or "costeffectiveness analys#s"	Expanders - Apply equivalent subjects<br>Search modes - Find all my search terms	Interface - EBSCOhost Research Databases<br>Search Screen - Advanced Search<br>Database - CINAHL with Full Text	8,714
S39	TI (cost or economic*) AND AB (costs or cost-effectiveness or markov)	Expanders - Apply equivalent subjects<br>Search modes - Find all my search terms	Interface - EBSCOhost Research Databases<br>Search Screen - Advanced Search<br>Database - CINAHL with Full Text	7,182
S40	S33 OR S34 OR S35 OR S36 OR S37 OR S38 OR S39	Expanders - Apply equivalent subjects<br>Search modes - Find all my search terms	Interface - EBSCOhost Research Databases<br>Search Screen - Advanced Search	64,551

			Database - CINAHL with Full Text	
S41	S22 AND S30 AND S40	Expanders - Apply equivalent subjects<br>Search modes - Find all my search terms	Interface - EBSCOhost Research Databases<br>Search Screen - Advanced Search<br>Database - CINAHL with Full Text	78
S42	TI (cost# N1 (illness or disease or sickness)) OR AB (cost# N1 (illness or disease or sickness))	Expanders - Apply equivalent subjects<br>Search modes - Find all my search terms	Interface - EBSCOhost Research Databases<br>Search Screen - Advanced Search<br>Database - CINAHL with Full Text	322
S43	TI (burden# N1 (illness or disease# or condition# or economic*)) OR AB (burden# N1 (illness or disease# or condition# or economic*))	Expanders - Apply equivalent subjects<br>Search modes - Find all my search terms	Interface - EBSCOhost Research Databases<br>Search Screen - Advanced Search<br>Database - CINAHL with Full Text	3,626
S44	TI ("quality-adjusted life years" or "quality adjusted life years" or QALY#) OR AB ("quality-adjusted life years" or "quality adjusted life years" or QALY#)	Expanders - Apply equivalent subjects<br>Search modes - Find all my search terms	Interface - EBSCOhost Research Databases<br>Search Screen - Advanced Search<br>Database - CINAHL with Full Text	1,055
S45	(MH "Quality-Adjusted Life Years")	Expanders - Apply equivalent subjects<br>Search modes - Find all my search terms	Interface - EBSCOhost Research Databases<br>Search Screen - Advanced Search<br>Database - CINAHL with Full Text	1,051
S46	(MH "Economic Aspects of Illness")	Expanders - Apply equivalent subjects<br>Search modes - Find all my search terms	Interface - EBSCOhost Research Databases<br>Search Screen - Advanced Search<br>Database - CINAHL with Full Text	2,562
S47	(MH "Health Care Costs+")	Expanders - Apply equivalent subjects	Interface - EBSCOhost Research Databases	16,519

Internal

S48		Search modes - Find all my search terms	Search Screen - Advanced Search<br>Database - CINAHL with Full Text	
S49	(MH "Costs and Cost Analysis")	Expanders - Apply equivalent subjects<br>Search modes - Find all my search terms	Interface - EBSCOhost Research Databases<br>Search Screen - Advanced Search<br>Database - CINAHL with Full Text	3,807
S50	TI (out-of-pocket N1 (payment# or expenditure# or cost# or spending or expense#)) OR AB (out-of-pocket N1 (payment# or expenditure# or cost# or spending or expense#))	Expanders - Apply equivalent subjects<br>Search modes - Find all my search terms	Interface - EBSCOhost Research Databases<br>Search Screen - Advanced Search<br>Database - CINAHL with Full Text	812
S51	TI (expenditure# N2 (health or direct or indirect)) OR AB (expenditure# N2 (health or direct or indirect))	Expanders - Apply equivalent subjects<br>Search modes - Find all my search terms	Interface - EBSCOhost Research Databases<br>Search Screen - Advanced Search<br>Database - CINAHL with Full Text	1,140
S52	TI ((adjusted or quality-adjusted) N1 year#) OR AB ((adjusted or quality-adjusted) N1 year#)	Expanders - Apply equivalent subjects<br>Search modes - Find all my search terms	Interface - EBSCOhost Research Databases<br>Search Screen - Advanced Search<br>Database - CINAHL with Full Text	1,899
S53	S42 OR S43 OR S44 OR S45 OR S46 OR S47 OR S48 OR S49 OR S50 OR S51	Expanders - Apply equivalent subjects<br>Search modes - Find all my search terms	Interface - EBSCOhost Research Databases<br>Search Screen - Advanced Search<br>Database - CINAHL with Full Text	27,072
	S22 AND S30 AND S52	Expanders - Apply equivalent subjects<br>Search modes - Find all my search terms	Interface - EBSCOhost Research Databases<br>Search Screen - Advanced Search	40

Internal

			Database - CINAHL with Full Text	
S54	S32 OR S41 OR S53	Expanders - Apply equivalent subjects<br>Search modes - Find all my search terms	Interface - EBSCOhost Research Databases<br>Search Screen - Advanced Search<br>Database - CINAHL with Full Text	216

Internal

Psycinfo

ProQuest

Search Strategy from ProQuest
2023 February 09 18:08

SEARCH STRATEGY

Set No.	Searched for	Databases	Results
S1	noft(DE "Coronavirus" OR DE "Middle East Respiratory Syndrome" OR DE "Severe Acute Respiratory Syndrome")	APA PsycInfo®	1087
S2	noft(DE "Coronavirus" OR DE "Middle East Respiratory Syndrome" OR DE "Severe Acute Respiratory Syndrome") AND la.exact("ENG")	APA PsycInfo®	954
S3	(noft(TX) ((noft(corona*) OR noft(corono*)) noft(n1) (noft(virus*) OR noft(viral*) OR noft(virinae*)))) AND la.exact("ENG")	APA PsycInfo®	0
S4	TX (coronavirus* or coronovirus* or coronavirinae* or Coronavirus* or Coronovirus* or Wuhan* or Hubei* or Huanan or "2019-nCoV" or 2019nCoV or nCoV2019 or "nCoV-2019" or "COVID-19" or COVID19 or "CORVID-19" or CORVID19 or "WN-CoV" or WNCoV or "HCoV-19" or HCoV19 or CoV or "2019 novel*" or Ncov or "n-cov" or "SARS-CoV-2" or "SARSCoV-2" or "SARSCoV2" or "SARS-CoV2" or SARSCov19 or "SARS-Cov19" or "SARSCov-19" or "SARS-Cov-19" or Ncovor or Ncorona* or Ncorono* or NcovWuhan* or NcovHubei* or NcovChina*	APA PsycInfo®	10

	or NcovChinese*)		
S5	SU.exact("SEVERE ACUTE RESPIRATORY SYNDROME")	APA PsycInfo®	413
S6	noft(TX) ((noft(corona*) or noft(corono*)) noft(n1) (noft(virus*) or noft(viral*) or noft(virinae*)))	APA PsycInfo®	0
S7	S2 OR S3 OR S4 OR S5 OR S6	APA PsycInfo® These databases are searched for part of your query.	1335
S8	noft("vaccination*")	APA PsycInfo®	6571
S9	noft("immunization*")	APA PsycInfo®	7772
S10	noft("immunisation*")	APA PsycInfo®	337
S11	noft("immunize*")	APA PsycInfo®	820
S12	noft("immunise*")	APA PsycInfo®	50
S13	noft("vaccine*")	APA PsycInfo®	7389
S14	noft("shots*")	APA PsycInfo®	1067
S15	S8 OR S9 OR S10 OR S11 OR S12 OR S13 OR S14	APA PsycInfo® These databases are searched for part of your query.	13186
S16	(noft(DE "Economics")) OR (noft(DE "Health Care Economics")) OR (noft(DE "Costs and Cost Analysis") OR noft(DE "Budgets") OR noft(DE "Health Care Costs")) OR noft(TI) (noft(economic*) OR noft(cost) OR noft(Costs) OR noft(costly) OR noft(costing) OR noft(price) OR noft(prices) OR noft(pricing) OR noft(pharmacoeconomic*)) OR noft(AB) (noft(economic*) OR noft(cost) OR noft(Costs) OR noft(costly) OR noft(costing) OR noft(price) OR noft(prices) OR noft(pricing) OR noft(pharmacoeconomic*))	APA PsycInfo®	9673

S17	S7 AND S15 AND S16	APA PsycInfo®<br>These databases are searched for part of your query.	1
S18	noft(MM "Economics")	APA PsycInfo®	60
S19	(noft(MM "Costs and Cost Analysis") OR noft(MM "Budgets") OR noft(MM "Cost Containment") OR noft(MM "Health Care Costs"))	APA PsycInfo®	39
S20	noft(TX) (noft(economic N1 model*))	APA PsycInfo®	0
S21	TI ("cost minimi*" or cost-utilit* or "health utilit*" or "economic evaluation*" or "economic review*" or "cost outcome" or "cost analys#s" or "economic analys#s" or "budget* impact analys#s") OR AB ("cost minimi*" or cost-utilit* or "health utilit*" or "economic evaluation*" or "economic review*" or "cost outcome" or "cost analys#s" or "economic analys#s" or "budget* impact analys#s")	APA PsycInfo®	7
S22	noft(TI cost-effective*) or noft(pharmacoeconomic*) or noft(pharmaco-economic*) or noft(cost-benefit) or noft(costs)	APA PsycInfo®	126874
S23	noft(AB "life year") or noft("life years") or noft(qaly*) or noft("cost-benefit analys#s") or noft("costeffectiveness analys#s")	APA PsycInfo®	3025
S24	((SU.exact("BENEFIT COST ANALYSIS") OR SU.exact("COST BENEFIT ANALYSIS") OR SU.exact("COST BENEFIT ANALYSIS") OR SU.exact("COST BENEFIT ANALYSES") OR	APA PsycInfo®	739

	SU.exact("COST BENEFIT ANALYSIS 03601") OR SU.exact("COST BENEFIT ANALYSES") OR SU.exact("COST BENEFIT ANALYSIS")) AND SU.exact("QUALITY ADJUSTED LIFE YEARS"))		
S25	S18 OR S19 OR S20 OR S21 OR S22 OR S23 OR S24	APA PsycInfo®<br>These databases are searched for part of your query.	127884
S26	S7 AND S15 AND S25	APA PsycInfo®<br>These databases are searched for part of your query.	1
S27	noft(TI) (noft(cost# N1) (noft(illness) or noft(disease) or noft(sickness))) OR noft(AB) (noft(cost# N1) (noft(illness) or noft(disease) or noft(sickness)))	APA PsycInfo®	0
S28	TI (burden# N1 (illness or disease# or condition# or economic*)) OR AB (burden# N1 (illness or disease# or condition# or economic*))	APA PsycInfo®	0
S29	TI ("quality-adjusted life years" or "quality adjusted life years" or QALY#) OR AB (	APA PsycInfo®	10

	"quality-adjusted life years" or "quality adjusted life years" or QALY#)		
S30	noft(DE "Health Care Economics")	APA PsycInfo®	111
S31	noft(DE "Costs and Cost Analysis")	APA PsycInfo®	1202
S32	noft(DE "Health Care Costs")	APA PsycInfo®	934
S33	noft(TI) (noft(out-of-pocket N1) (noft(payment#) or noft(expenditure#) or noft(cost#) or noft(spending) or noft(expense#))) OR noft(AB) (noft(out-of-pocket N1) (noft(payment#) or noft(expenditure#) or noft(cost#) or noft(spending) or noft(expense#)))	APA PsycInfo®	0
S34	TI (expenditure# N2 (health or direct or indirect)) OR AB (expenditure# N2 (health or direct or indirect))	APA PsycInfo®	0
S35	TI ((adjusted or quality-adjusted) N1 year#) OR AB ((adjusted or quality-adjusted) N1 year#)	APA PsycInfo®	0
S37	S27 OR S28 OR S29 OR S30 OR S31 OR S32 OR S33 OR S34 OR S35	APA PsycInfo®<br>These databases are searched for part of your query.	1966
S38	S7 AND S15 AND S37	APA PsycInfo®<br>These databases are searched for part of your query.	0
S39	S17 OR S26 OR S38	APA PsycInfo®<br>These databases are searched for part of your query.	2

Database copyright Ó 2023 ProQuest LLC. All rights reserved.

Terms and Conditions Contact ProQuest

MEDLINE

Medline via Ovid (08 February 2023)

Title: The economic evaluation and cost benefit analysis for COVID-19 vaccination

Search terms		Items found
Population: COVID-19[1)]		
1	Exp Coronavirus/	160,105
2	Exp Coronavirus Infections/	221,170
3	(COVID* or sars cov* or sarscov* or corona virus or coronavirus or<br>ncov).ab,kf,ti	321,518
4	1 or 2 or 3	335,987
Intervention: Vaccination (limit to COVID-19 search)		
5	Vaccine	1,075
6	Vaccination	4,079
7	Vaccin	45
8	Immunization	219
9	5 or 6 or 7 or 8	5,413
Health economic aspects /Economic aspects[2)]		
10	(economics/ or exp "costs and cost analysis"/ or economics, dental/ or exp "economics, hospital"/ or economics, medical/ or economics, nursing/ or economics, pharmaceutical/ or (economic$ or cost or costs or costly or costing or price or prices or pricing or pharmacoeconomic$).ti,ab. or (expenditure$ not energy).ti,ab. or (value adj1 money).ti,ab. or budget$.ti,ab.) not (((energy or oxygen) adj cost) or (metabolic adj cost) or ((energy or oxygen) adj<br>expenditure)).ti,ab. not (letter or historical article).pt.	1,119,021
Combined sets		
11	4 and 9 and 10	364
Health economic aspects /Economic aspects[2)]		
12	*economics/	10,804
13	exp *"costs and cost analysis"/	79,232
14	(economic adj2 model*).mp.	14,723
15	(cost minimi* or cost-utilit* or health utilit* or economic evaluation* or economic<br>review* or cost outcome or cost analys?s or economic analys?s or budget* impact<br>analys?s).ti,ab,kf,kw.	39,500
16	(cost-effective* or pharmacoeconomic* or pharmaco-economic* or cost-benefit<br>or costs).ti,kf,kw	84,434
17	(life year or life years or qaly* or cost-benefit analys?s or cost-effectiveness<br>analys?s).ab,kf,kw	37,308
18	(cost or economic*).ti,kf,kw. and (costs or cost-effectiveness or markov).ab.	69,185
19	12 or 13 or 14 or 15 or 16 or 17 or 18	205,732

Combined sets		
20	4 and 9 and 19	41
Health economic aspects /Economic aspects[2)]		
21	(cost? adj2 (illness or disease or sickness)).tw.	4,685
22	(burden? adj2 (illness or disease? or condition? or economic*)).tw.	51,411
23	("quality-adjusted life years" or "quality adjusted life years" or QALY?).tw.	15,858
24	Quality-adjusted life years (limit to COVID-19 search)	98
25	"cost of illness"/	31,243
26	Health expenditures/	23,630
27	(out-of-pocket adj2 (payment? or expenditure? or cost? or spending or expense?)).tw.	6,351
28	(expenditure? adj3 (health or direct or indirect)).tw.	10,405
29	((adjusted or quality-adjusted) adj2 year?).tw.	27,167
30	21 or 22 or 23 or 24 or 25 or 26 or 27 or 28 or 29	132,187
Combined sets		
31	4 and 9 and 30	73
32	11 or 20 or 31	407

1) COVID-19 and pregnancy (sbu.se)
2) Bilaga 1 Sökdokumentation (sbu.se)

.ab. =Abstract

.ab,ti. = Abstract or title

.af.= All fields

.bt.= Book title

Exp= Term from the Medline controlled vocabulary, including terms found below this term in the MeSH hierarchy

.kf.= Keyword heading word

.sh.= Term from the Medline controlled vocabulary

.ti. = Title

/ = Term from the Medline controlled vocabulary, but does not include terms found below this term in the MeSH hierarchy

* = Focus (if found in front of a MeSH-term)

* or $= Truncation (if found at the end of a free text term)

.mp=text, heading word, subject area node,

title “ “ = Citation Marks; searches for an

exact phrase

ADJn= positional operator that lets you retrieve records that contain your terms (in any order) within a specified number (n) of words of each other.

Appendix 2

Excluded studies

Articles that seemed relevant based on their titles and abstracts, but were excluded based on their full texts, as they did not meet the inclusion criteria.

Internal

Article (author, title, source)	Main reason for exclusion
Letter from the Editor. Human Vaccines and Immunotherapeutics. 2020;16(9):2003-4.	Not Economic evaluation
Meeting of the Immunization and Vaccine-related Implementation Research Advisory Committee (IVIR-AC), March 20212021 2021-4-30. World Health Organization.	Not Economic evaluation
3.H. Workshop: The role of HTA for COVID-19 vaccines: present and future perspectives...14th European Public Health Conference (Virtual), Public health futures in a changing world, November 10-12, 2021. European Journal of Public Health. 2021;31:iii75-iii.	Not Economic evaluation
Abraham I, Lee KKC, Gregg M. Journal of Medical Economics in review: the best of 2021. Journal of Medical Economics. 2022;25(1):282-6.	Wrong Population
Addae A, Ramjee L, Tremblay G. EE7 Economic Evaluation of COVID-19 Vaccines: A Targeted Literature Review. Value in Health. 2022;25(7):S336.	Wrong Publication text
Agarwal RN, Aggarwal R, arapu P, Aggarwal H, Verma A, Haque A, et al. COVID-19 Vaccination Drive in a Low-Volume Primary Care Clinic: Challenges & Lessons Learned in Using Homegrown Self-Scheduling Web-Based Mobile Platforms. Vaccines. 2022;10(7).	Not Economic evaluation
Al-M, hari A, Brennan RJ, Abubakar A, Hajjeh R. Towards healthier and better prepared Eastern Mediterranean Region: Moving forward post-COVID-19. BMJ Global Health. 2022;7(4).	Not Economic evaluation
Ale BJM, Slater DH, Hartford DND. The ethical dilemmas of risky decisions. Risk analysis : an official publication of the Society for Risk Analysis. 2022.	Not Economic evaluation
Alvarez MM, Bravo-González S, Trujillo-De Santiago G. Modeling vaccination strategies in an Excel spreadsheet: Increasing the rate of vaccination is more effective than increasing the vaccination coverage for containing COVID-19. PLoS ONE. 2021;16(7).	Not Economic evaluation
Angelis A, Baltussen R, Tervonen T. The Need for Novel Approaches in Assessing the Value of COVID-19 Vaccines. American Journal of Public Health. 2021;111(2):205-8.	Not Economic evaluation

Appleby J. Will COVID-19 vaccines be cost effective - And does it matter? The BMJ. 2020;371.	Wrong study design
Appleby J. The public finance cost of COVID-19. The BMJ. 2022;376.	Wrong study design
Araja D, Berkis U, Lunga A, Murovska M. PMU29 Burden of COVID-19 Consequences: an Example of Post-viral Chronic Fatigue Syndrome. Value in Health. 2021;24:S149-S50.	Not Economic evaluation
Asukai Y, Briggs A, Garrison LP, Geisler BP, Neumann PJ, Ollendorf DA. Principles of Economic Evaluation in a Pandemic Setting: An Expert Panel Discussion on Value Assessment During the Coronavirus Disease 2019 Pandemic. PharmacoEconomics. 2021;39(11):1201-8.	Wrong Intervention
Bartsch SM, O'Shea KJ, Chin KL, Strych U, Ferguson MC, Bottazzi ME, et al. Maintaining face mask use before and after achieving different COVID-19 vaccination coverage levels: a modelling study. The Lancet Public Health. 2022;7(4):e356-e65.	Wrong Intervention
Basile M, Di Brino E, Rumi F, Cicchetti A. The Cost-Effectiveness Of The Anti-COVID Vaccination Campaign In The Italian Healthcare Setting. International Journal of Technology Assessment in Health Care. 2022;38:S68.	Wrong Population
Beck E, Biundo E, Devlin N, Doherty TM, Garcia-Ruiz AJ, Postma M, et al. Capturing the value of vaccination within health technology assessment and health economics: Literature review and novel conceptual framework. Vaccine. 2022;40(30):4008-16.	Not Economic evaluation
Benest J, Rhodes S, Quaife M, Evans TG, White RG. Optimising vaccine dose in inoculation against SARS-CoV-2, a multi-factor optimisation modelling study to maximise vaccine safety and efficacy. Vaccines. 2021;9(2):1-15.	Not Economic evaluation
Benner J, Adair N, Friedman M, Menzin J, Sussman M. PIN153 Lessons Learned from Economic Models of Influenza Vaccines and Applications to SARS-CoV-2. Value in Health. 2020;23:S569.	Wrong Population
Berry DA, Berry S, Hale P, Isakov L, Lo AW, Siah KW, et al. A cost/benefit analysis of clinical trial designs for COVID-19 vaccine candidates. PLoS ONE. 2021;15(12).	Wrong Population

Blank C. Pharmacists Central to Distributing Pediatric COVID-19 Vaccines. Drug Topics. 2022;166(1):10-1.	Wrong Publication text
Bloom DE, Cadarette D, Ferranna M. The Societal Value of Vaccination in the Age of COVID-19. American Journal of Public Health. 2021;111(6):1049-54.	Not Economic evaluation
Boersma C, Postma MJ. Health Economics of Vaccines: From Current Practice to Future Perspectives. Value in Health. 2021;24(1):1-2.	Not Economic evaluation
Bollyky TJ, Murray CJL, Reiner RC. Epidemiology, not geopolitics, should guide COVID-19 vaccine donations. The Lancet. 2021;398(10295):97-9.	Not Economic evaluation
Bortz RH, Florez C, Laudermilch E, Wirchnianski AS, Lasso G, Malonis RJ, et al. Single-Dilution COVID-19 Antibody Test with Qualitative and Quantitative Readouts. mSphere. 2021;6(2):201-2.	Not Economic evaluation
Bowie C, Friston K. A 12-month projection to September 2022 of the COVID-19 epidemic in the UK using a dynamic causal model. Front. 2022;10:999210.	Not Economic evaluation
Braga LM, da Motta OJR, Gomes AP, Oliveira PC, Vicari MV, Siqueira-Batista R. SARS-CoV-2 transmission by aerosols: An underestimated question? Revista da Associacao Medica Brasileira. 2021;67:5-7.	Wrong Intervention
Brassel S, Neri M, Steuten L. PIN62 Recognising the broader value of vaccines in HTA: ready for prime time? Value in Health. 2021;24:S117.	Not Economic evaluation
Brophy JM. SARS-CoV-2 testing in travellers: Can we be smarter? CMAJ. 2022;194(1):E20.	Wrong Intervention
Cadeddu C, Ricciardi W. The role of HTA in COVID-19 vaccination campaign: the Italian experience...14th European Public Health Conference (Virtual), Public health futures in a changing world, November 10-12, 2021. European Journal of Public Health. 2021;31:iii76-iii.	Not Economic evaluation

Campos-Mercade P, Meier AN, Schneider FH, Meier S, Pope D, Wengström E. Monetary incentives increase COVID-19 vaccinations. Science. 2021;374(6569):879-82.	Not Economic evaluation
Chang AY, Aaby P, Avidan MS, Benn CS, Bertozzi SM, Blatt L, et al. One vaccine to counter many diseases? Modelling the economics of oral polio vaccine against child mortality and COVID-19. 2022.	Wrong Population
Chhatwal J, Postma MJ. Health Economics of Interventions to Tackle the Coronavirus 2019 Pandemic. Value in Health. 2021;24(5):605-6.	Not Economic evaluation
Chua BWB, Huynh VA, Lou J, Goh FT, Clapham H, Teerawattananon Y, et al. Protocol for the economic evaluation of COVID-19 pandemic response policies. BMJ Open. 2021;11(9).	Wrong Country
Chumakov K, Avidan MS, Benn CS, Bertozzi SM, Blatt L, Chang AY, et al. Old vaccines for new infections: Exploiting innate immunity to control COVID-19 and prevent future pandemics. Proceedings of the National Academy of Sciences of the United States of America. 2021;118(21).	Not Economic evaluation
Darden ME, Dowdy D, Gardner L, Hamilton BH, Kopecky K, Marx M, et al. Modeling to inform economy-wide pandemic policy: Bringing epidemiologists and economists together. Health Economics (United Kingdom). 2022;31(7):1291-5.	Not Economic evaluation
DeGruttola V, Goyal R, Martin NK, Wang R. Network methods and design of randomized trials: Application to investigation of COVID-19 vaccination boosters. Clinical Trials. 2022;19(4):363-74.	Not Economic evaluation
Dobie T, Zirlinger M. Welcome all. Neuron. 2021;109(19):3013-4.	Wrong Publication text
Donaldson C, Biosca O. COVID-19: An economic perspective on vaccinating the world. The BMJ. 2021;373.	Not Economic evaluation
Dunn R, Caruana DL, Dhuper S, Szema AM. Cost-Benefit Analysis of a Private COVID-19 Vaccination Center. American Journal of Respiratory and Critical Care Medicine. 2022;205(1).	Wrong Population

Elvidge J, Dawoud D. Assessing Technologies for COVID-19: What are the Challenges for Health Technology Assessment Agencies? Findings From a Survey and Roundtable Workshop. PharmacoEconomics. 2021;39(12):1455-63.	Not Economic evaluation
Emanuel EJ, Osterholm M, Gounder CR. A National Strategy for the "new Normal" of Life with COVID. JAMA. 2022;327(3):211-2.	Not Economic evaluation
Engebretsen S, Palomares ADL, Rø G, Kristoffersen AB, Lindstrøm JC, Engø-Monsen K, et al. A real-time regional model for COVID-19: Probabilistic situational awareness and forecasting. PLoS Computational Biology. 2023;19(1).	Not Economic evaluation
epitte S, Alleman T, Nopens I, Baetens J, Coenen S, De Smedt D. Cost-Effectiveness of COVID-19 Policy Measures: A Systematic Review. Value in Health. 2021;24(11):1551-69.	Wrong Intervention
ey D, Garg PK. Do we need evidence for evidence-based medicine? Journal of Evaluation in Clinical Practice. 2022;28(5):731-2.	Not Economic evaluation
Fabiani M, Mateo-Urdiales A, Sacco C, Rota MC, Petrone D, Bressi M, et al. Relative effectiveness of a 2nd booster dose of COVID-19 mRNA vaccine up to four months post administration in individuals aged 80 years or more in Italy: A retrospective matched cohort study. Vaccine. 2023;41(1):76-84.	Not Economic evaluation
Far, a D, Alberti T. Modeling the second wave of COVID-19 infections in France and Italy via a stochastic SEIR model. Chaos (Woodbury, NY). 2020;30(11):111101.	Not Economic evaluation
Ferreira LS, Canton O, da Silva RLP, Poloni S, Sudbrack V, Borges ME, et al. Assessing the best time interval between doses in a two-dose vaccination regimen to reduce the number of deaths in an ongoing epidemic of SARS-CoV-2. PLoS Computational Biology. 2022;18(3).	Not Economic evaluation
Fox N, Adams P, Grainger D, Herz J, Austin C. The Value of Vaccines: A Tale of Two Parts. Vaccines. 2022;10(12).	Not Economic evaluation
Gibson S, Saunders R, Stasko N, Bickerstaff CB, Oakley J, Osterman M, et al. Economic and clinical impact of a novel, light-based, at-home antiviral treatment on mild-to-moderate COVID-19. Journal of Medical Economics. 2022;25(1):503-14.	Wrong Intervention

Giubilini A, Savulescu J, Wilkinson D. Queue questions: Ethics of COVID-19 vaccine prioritization. Bioethics. 2021;35(4):348-55.	Not Economic evaluation
Golladay GJ. New World Order. Arthroplasty Today. 2021;7:242.	Not Economic evaluation
Götz G, Herold D, Klotz PA, Schäfer JT. Efficiency in COVID-19 vaccination campaigns—a comparison across germany's federal states. Vaccines. 2021;9(7).	Not Economic evaluation
Greaves F, Boysen M. NICE's approach to measuring value. The BMJ. 2021;372.	Not Economic evaluation
Green RJ, Mustafa F. Guest editorial. Current Allergy and Clinical Immunology. 2020;33(3):128.	Not Economic evaluation
Griesenbach U, Yáñez-Muñoz RJ. The British Society for Gene and Cell Therapy. Human Gene Therapy. 2021;32(19):983-5.	Not Economic evaluation
Guan WJ, Zhong NS. Reply. Respirology. 2020;25(8):899.	Not Economic evaluation
Gupta A, Kunte R, Goyal N, Ray S, Singh K. A comparative analysis of control measures on-board ship against COVID-19 and similar novel viral respiratory disease outbreak: Quarantine ship or disembark suspects? Medical Journal Armed Forces India. 2021;77:S430-S6.	Not Economic evaluation
Guttieres D, Sinskey A, Springs S. Models to inform neutralizing antibody therapy strategies during pandemics: The case of SARS-CoV-2. Antibody Therapeutics. 2021;4(1):60-71.	Wrong Intervention
Hageman JR, Alkureishi LA. The status of the COVID-19 vaccines for children age 5 to 11 years. Pediatric Annals. 2021;50(11):e444-e5.	Not Economic evaluation

Hagens A, İnkaya AÇ, Yildirak K, Sancar M, van der Schans J, Acar Sancar A, et al. COVID-19 vaccination scenarios: A cost-effectiveness analysis for turkey. Vaccines. 2021;9(4).	Wrong Country
Hodes S, Kovacevic L. Primary care networks: Where are we? Where is the evidence base? What might the future bring? The BMJ. 2021;375.	Not Economic evaluation
Hodes S, Stanley S, Majeed A. A national vaccination service for the NHS in England: A proposal to be considered with caution. The BMJ. 2022;376.	Not Economic evaluation
Hudson R, Clark-Wright J, Saunders E, Akhtar T, Carroll S. PNS142 What Difference Would a Wider Definition of Prevention Make to Decisions Reached By the Joint Committee on Vaccination and Immunisation (JCVI)? Value in Health. 2020;23:S664.	Wrong Population
Ibrahim D, Kis Z, Tak K, Papathanasiou MM, Kontoravdi C, Chachuat B, et al. Model-based planning and delivery of mass vaccination campaigns against infectious disease: Application to the COVID-19 pandemic in the UK. Vaccines. 2021;9(12).	Not Economic evaluation
Jahn B, Sroczynski G, Bicher M, Rippinger C, Mühlberger N, Santamaria J, et al. POSA172 How to Optimize COVID-19 Vaccination Considering Limited Vaccination Capacities: A Decision-Analytic Framework and Modeling Study. Value in Health. 2022;25(1):S121.	Not Economic evaluation
Jour A. Was EU's COVID-19 vaccine procurement strategy irrational? A re-analysis based on cost-effectiveness considerations. BMC health services research. 2022;22(1):1410.	Wrong Population
Jour A. Cost-effectiveness of future lockdown policies against the COVID-19 pandemic. Health Services Management Research. 2022.	Wrong Intervention
Juneau CE, Pueyo T, Bell M, Gee G, Collazzo P, Potvin L. Lessons from past pandemics: a systematic review of evidence-based, cost-effective interventions to suppress COVID-19. Systematic Reviews. 2022;11(1).	Not Economic evaluation
Kabir M, Saqib MAN, Zaid M, Ahmed H, Afzal MS. COVID-19, economic impact and child mortality: A global concern. Clinical Nutrition. 2020;39(7):2322-3.	Not Economic evaluation

Kane MMD. Effects of the COVID-19 Pandemic on Well-Child Care and Recommendations for Remediation. Pediatric Annals. 2021;50(12):e488-e93.	Not Economic evaluation
Kaufman J, Bagot KL, Hoq M, Leask J, Seale H, Biezen R, et al. Factors influencing australian healthcare workers' COVID-19 vaccine intentions across settings: A cross-sectional survey. Vaccines. 2022;10(1).	Not Economic evaluation
Kelly SL, Le Rutte EA, Richter M, Penny MA, Shattock AJ. COVID-19 vaccine booster strategies in light of emerging viral variants: Frequency, timing, and target groups. 2022.	Not Economic evaluation
Khatami SN, Gopalappa C. Deep reinforcement learning framework for controlling infectious disease outbreaks in the context of multi-jurisdictions. 2022.	Wrong Intervention
Kohns Vasconcelos M, Marazia C, Koniordou M, Fangerau H, Drexler I, Afum-Adjei Awuah A. A conceptual approach to the rationale for SARS-CoV-2 vaccine allocation prioritisation. Pathogens and Global Health. 2021;115(5):273-6.	Not Economic evaluation
Korkmaz E, Balmert SC, Carey CD, Erdos G, Falo LD. Emerging skin-targeted drug delivery strategies to engineer immunity: A focus on infectious diseases. Expert Opinion on Drug Delivery. 2021;18(2):151-67.	Not Economic evaluation
Kostoff RN, Calina D, uc D, Briggs MB, Vlachoyiannopoulos P, Svistunov AA, et al. Why are we vaccinating children against COVID-19? Toxicology Reports. 2021;8:1665-84.	Not Economic evaluation
Kulcar V, Straganz C, Kreh A, Siller H, File N, Canazei M, et al. University students' adherence and vaccination attitudes during the COVID-19 pandemic: Focusing on costs and benefits. Applied Psychology Health and Well-being. 2022;14(2):572-90.	Not Economic evaluation
Kyrychko YN, Blyuss KB, Brovchenko I. Mathematical modelling of the dynamics and containment of COVID-19 in Ukraine. Sci. 2020;10(1):19662.	Wrong Intervention
Lau JWY. Editor's Perspective September 2022. International Journal of Surgery. 2022;105.	Not Economic evaluation

Lee BE, Sikora C, Faulder D, Risling E, Little LA, Qiu Y, et al. Early warning and rapid public health response to prevent COVID-19 outbreaks in long-term care facilities (LTCF) by monitoring SARS-CoV-2 RNA in LTCF site-specific sewage samples and assessment of antibodies response in this population: Prospective study protocol. BMJ Open. 2021;11(8).	Not Economic evaluation
Leong S, Eom K, Ishii K, Aichberger MC, Fetz K, Müller TS, et al. Individual costs and community benefits: Collectivism and individuals' compliance with public health interventions. PLoS ONE. 2022;17(11).	Not Economic evaluation
Lewis D. What scientists have learnt from COVID lockdowns. Nature. 2022;609(7926):236-9.	Not Economic evaluation
Liu Y, mann FG, Barnard RC, Pearson CAB, Group CC-W, Pastore R, et al. Optimising health and economic impacts of COVID-19 vaccine prioritisation strategies in the WHO European Region. MedRxiv : the Preprint Server for Health Sciences. 2021;14:14.	Not Economic evaluation
López F, Català M, Prats C, Estrada O, Oliva I, Prat N, et al. A Cost–Benefit Analysis of COVID-19 Vaccination in Catalonia. Vaccines. 2022;10(1).	Wrong Population
Lorgelly PK, Adler A. Impact of a Global Pandemic on Health Technology Assessment. Applied Health Economics and Health Policy. 2020;18(3):339-43.	Not Economic evaluation
Luyten J, Kessels R. Stability of Stated Preferences: Vaccine Priority Setting before and during the First COVID-19 Lockdown. Medical decision making : an international journal of the Society for Medical Decision Making. 2023:272989X221150185.	Not Economic evaluation
MacIntyre CR. Navigating post-vaccine COVID-19 futures in the health and economic context. The Lancet Infectious Diseases. 2021;21(7):893-4.	Not Economic evaluation
Makhoul M, Abu-Hijleh F, Ayoub HH, Seedat S, Chemaitelly H, Abu-Raddad LJ. Modeling the population-level impact of treatment on COVID-19 disease and SARS-CoV-2 transmission. Epidemics. 2022;39.	Wrong Intervention
Makhoul M, Ayoub HH, Chemaitelly H, Seedat S, Mumtaz GR, Al-Omari S, et al. Epidemiological impact of sars-cov-2 vaccination: Mathematical modeling analyses. Vaccines. 2020;8(4):1-16.	Not Economic evaluation

Maltezou HC, Giannouchos TV, Pavli A, Tsonou P, Dedoukou X, Tseroni M, et al. Costs associated with COVID-19 in healthcare personnel in Greece: a cost-of-illness analysis. Journal of Hospital Infection. 2021;114:126-33.	Wrong Population
Mann FG, Davies NG, Vassall A, Edmunds WJ, Jit M, Centre for the Mathematical Modelling of Infectious Diseases C-wg. The potential health and economic value of SARS-CoV-2 vaccination alongside physical distancing in the UK: a transmission model-based future scenario analysis and economic evaluation. The Lancet Infectious Diseases. 2021;21(7):962-74.	Wrong Population
Mar J, Ibarrondo O, Larrañaga I, Aguiar M, Stollenverk N, Bidaurrazaga J, et al. P1 Cost-Utility Analysis of COVID-19 Vaccines in the Basque Country in the First Semester of 2021. Value in Health. 2022;25(12):S1.	Wrong Publication text
Marchetti S, Borin A, Conteduca FP, Ilardi G, Guzzetta G, Poletti P, et al. An epidemic model for SARS-CoV-2 with self-adaptive containment measures. PLoS ONE. 2022;17(7).	Not Economic evaluation
McBryde ES, Meehan MT, Adegboye OA, Adekunle AI, Caldwell JM, Pak A, et al. Role of modelling in COVID-19 policy development. Paediatric Respiratory Reviews. 2020;35:57-60.	Not Economic evaluation
McCarthy CV, O'Mara O, van Leeuwen E, Group CC-W, Jit M, mann F. The impact of COVID-19 vaccination in prisons in England and Wales: a metapopulation model. BMC Public Health. 2022;22(1):1003.	Not Economic evaluation
McWhinnie D, Magalhães C, Philip B. IAAS Statement on the COVID pandemic. Ambulatory Surgery. 2021;27(3):48.	Wrong Publication text
Meyer RJ. Priority Review Vouchers: GAO Report Provides Scant Evidence of Success. Clinical and Translational Science. 2021;14(1):8-10.	Wrong study design
Miles DK, Heald AH, Stedman M. How fast should social restrictions be eased in England as COVID-19 vaccinations are rolled out? International Journal of Clinical Practice. 2021;75(7).	Wrong Intervention
Millar MR, Gourtsoyannis Y, Jayakumar A. Ethics of vaccination: Should capability measures be used to inform SARS-CoV-2 vaccination strategies? British Journal of Clinical Pharmacology. 2022;88(1):47-55.	Not Economic evaluation
Mintram K, Anagnostou A, Anokye N, Okine E, Groen D, Saha A, et al. CALMS: Modelling the long-term health and economic impact of COVID-19 using agent-based simulation. PLoS ONE. 2022;17(8).	Wrong Population

Minzenberg MJ, Yoon JH. A profile of high scores on the Peters delusion inventory and low engagement in pandemic-related health behaviors in a non-clinical sample. Schizophrenia Research. 2022;250:10-2.	Not Economic evaluation
Moore S, Hill EM, Dyson L, Tildesley MJ, Keeling MJ. Modelling optimal vaccination strategy for SARS-CoV-2 in the UK. PLoS Computational Biology. 2021;17(5):e1008849.	Not Economic evaluation
Moy N, Dulleck U, Shah A, Messmann H, Thrift AP, Talley NJ, et al. Risk-based decision-making related to preprocedural coronavirus disease 2019 testing in the setting of GI endoscopy: management of risks, evidence, and behavioral health economics. Gastrointestinal Endoscopy. 2022;96(5):735-42.e3.	Not Economic evaluation
Mungmunpuntipantip R, Wiwanitkit V. Expected cost effectiveness of the fourth dose of COVID-19 vaccine against the omicron variant of COVID-19: a preliminary report. International Journal of Physiology, Pathophysiology and Pharmacology. 2022;14(4):272-5.	Wrong Population
Nana-Kyere S, Seidu B. A Mathematical Evaluation of the Cost-Effectiveness of Self-Protection, Vaccination, and Disinfectant Spraying for COVID-19 Control. Genetics Research. 2022;2022.	Wrong Population
Naveel T, Chunda R. The Reported Side Effects of Corona Virus Vaccination among Oral Health Care Workers. Pakistan Journal of Medical and Health Sciences. 2022;16(3):546-51.	Not Economic evaluation
Neumann PJ, Cohen JT, Kim DD, Ollendorf DA. Consideration of value-based pricing for treatments and vaccines is important, even in the COVID-19 pandemic. Health Affairs. 2021;40(1):53-61.	Not Economic evaluation
Nurchis MC, Lontano A, Pascucci D, Sapienza M, Marziali E, Castrini F, et al. COVID-19 Vaccination Campaign among the Health Workers of Fondazione Policlinico Universitario Agostino Gemelli IRCCS: A Cost–Benefit Analysis. International Journal of Environmental Research and Public Health. 2022;19(13).	Wrong Population
Padula W, Malaviya S, Reid N, Chingcuanco F, Ballreich J, Tierce J, et al. PIN150 Economic VALUE of Treatment and Vaccine Technologies to Address the COVID-19 Pandemic: A Cost-Effectiveness and Budget IMPACT Analysis. Value in Health. 2020;23:S568.	Wrong Country
Paganini I, Sani C, Chilleri C, Baccini M, Antonelli A, Bisanzi S, et al. Assessment of the feasibility of pool testing for SARS-CoV-2 infection screening. Infectious Diseases. 2022;54(7):478-87.	Not Economic evaluation

Persad G, ya A. A Comprehensive COVID-19 Response - The Need for Economic Evaluation. New England Journal of Medicine. 2022;386(26):2449-51.	Not Economic evaluation
Persson U, Olofsson S, Althin R, Palmborg A, Dorange AC. Acceptance and application of a broad population health perspective when evaluating vaccine. Vaccine. 2022;40(24):3395-401.	Wrong Population
Pham TM, Westerhof I, Bootsma MCJ, Kretzschmar ME, Rozhnova G, Bruijning-Verhagen P. Seasonal patterns of SARS-CoV-2 transmission in secondary schools: a modelling study. 2022.	Not Economic evaluation
Phuong PT, Ha PN. Letter from Vietnam. Respirology. 2023;28(2):192-3.	Not Economic evaluation
Pitman R, Khurana P. EE540 Cost-Effectiveness of Vaccines Targeting Infectious Diseases - Has COVID-19 Changed the Landscape? Value in Health. 2022;25(12):S162.	Not Economic evaluation
Plans-Rubió P. The Cost Effectiveness of Stockpiling Drugs, Vaccines and Other Health Resources for Pandemic Preparedness. PharmacoEconomics - Open. 2020;4(3):393-5.	Wrong Intervention
Podolsky MI, Present I, Neumann PJ, Kim DD. A Systematic Review of Economic Evaluations of COVID-19 Interventions: Considerations of Non-Health Impacts and Distributional Issues. Value in Health. 2022;25(8):1298-306.	Wrong study design
Postma M, Boersma C, Hagens A, van der Schans J. The Evolving HTAi Statement on COVID-19 Vaccines...14th European Public Health Conference (Virtual), Public health futures in a changing world, November 10-12, 2021. European Journal of Public Health. 2021;31:iii75-iii.	Wrong Publication text
Postma MJ, Chhatwal J. COVID-19 Health Economics: Looking Back and Scoping the Future. Value in Health. 2022;25(5):695-6.	Not Economic evaluation
Razai MS, Oakeshott P, Esmail A, Wiysonge CS, Viswanath K, Mills MC. COVID-19 vaccine hesitancy: the five Cs to tackle behavioural and sociodemographic factors. Journal of the Royal Society of Medicine. 2021;114(6):295-8.	Not Economic evaluation

Razavi-Shearer D, Blach S, Estes C, Gamkrelidze I, Mooneyhan E, Murphy K, et al. Global hbv cascade of care: The pre-COVID-19 baseline. Hepatology. 2021;74:46A.	Not Economic evaluation
Richards F, Kodjamanova P, Chen X, Li N, Atanasov P, Bennetts L, et al. Economic Burden of COVID-19: A Systematic Review. ClinicoEconomics and Outcomes Research. 2022;14:293-307.	Wrong study design
Rodrigues CMC, Plotkin SA. Impact of Vaccines; Health, Economic and Social Perspectives. Frontiers in Microbiology. 2020;11.	Wrong Intervention
Rojas P, Epp C, Gozzo L, Geba D, Epstein M, Michel S. HTA66 Outcomes Examined in Clinical Trials of Medicines for COVID-19: A Review of Regulatory Agencies' Websites. Value in Health. 2022;25(7):S515-S6.	Not Economic evaluation
Rountree C, Prentice G. Segmentation of intentions towards COVID-19 vaccine acceptance through political and health behaviour explanatory models. Irish Journal of Medical Science. 2022;191(5):2369-83.	Not Economic evaluation
Rumi F, Basile M, Cicchetti A. Cost-effectiveness and budget impact analysis for high dose quadrivalent influenza vaccine in the Italian elderly population. Global and Regional Health Technology Assessment. 2021;8:105-13.	Wrong Intervention
Saadi N, Chi YL, Ghosh S, Eggo RM, McCarthy CV, Quaife M, et al. Models of COVID-19 vaccine prioritisation: a systematic literature search and narrative review. BMC Medicine. 2021;19(1):318.	Not Economic evaluation
Santos P, Almeida F. Exosome-Based Vaccines: History, Current State, and Clinical Trials. Frontiers in Immunology. 2021;12.	Not Economic evaluation
Savinkina A, Gonsalves G, Ross JS, Paltiel AD. Determining population-level allocation strategies for COVID-19 treatments in the United States using a quantitative framework, a case study using nirmatrelvir/ritonavir. 2022.	Wrong Intervention
Schulenburg JMG. COVID-19: not the time for health economists? A plea for more proactive health economic involvement. European Journal of Health Economics. 2021;22(7):1001-4.	Not Economic evaluation

Schüler L, Calabrese JM, Attinger S. Data driven high resolution modeling and spatial analyses of the COVID-19 pandemic in Germany. PLoS ONE. 2021;16(8).	Not Economic evaluation
Shaker M, Abrams EM, Greenhawt M. A Cost-Effectiveness Evaluation of Hospitalizations, Fatalities, and Economic Outcomes Associated with Universal Versus Anaphylaxis Risk-Stratified COVID-19 Vaccination Strategies. The Journal of Allergy & Clinical Immunology in Practice. 2021;9(7):2658-68.e3.	Wrong Population
Shaker M, Mauger D, Fuhlbrigge AL. Value-Based, Cost-Effective Care: The Role of the Allergist-Immunologist. Journal of Allergy and Clinical Immunology: In Practice. 2023;11(1):132-9.	Wrong Population
Sonabend R, Whittles LK, Imai N, Perez-Guzman PN, Knock ES, Rawson T, et al. Non-pharmaceutical interventions, vaccination, and the SARS-CoV-2 delta variant in England: a mathematical modelling study. Lancet. 2021;398(10313):1825-35.	Not Economic evaluation
Sookaromdee P, Wiwanitkit V. New COVID-19 Vaccines, Its Cost and Shelf Life: A Cost Effectiveness Analysis. Archives of Medical Research. 2021;52(4):453.	Wrong Population
Stevenson M, Metry A, Messenger M. Modelling of hypothetical sars-cov-2 point of care tests for routine testing in residential care homes: Rapid cost-effectiveness analysis. Health Technology Assessment. 2021;25(39):vi-73.	Wrong Intervention
Tafuri S, Bianchi FP, Stefanizzi P. The public health and the question of the "best vaccine". Vaccine. 2022;40(28):3813-4.	Not Economic evaluation
Tekerek B, Günaltay MM, Ozler G, Turgut M. Determinants of COVID-19 cases and deaths in OECD countries. Journal of Public Health (Germany). 2023.	Not Economic evaluation
The L. Lessons from the NHS for UHC and health security. The Lancet. 2021;397(10288):1859.	Not Economic evaluation
Thompson KM, Badizadegan K. Health economic analyses of secondary vaccine effects: a systematic review and policy insights. Expert Review of Vaccines. 2022;21(3):297-312.	Wrong study design

Tiirinki H, Viita-aho M, Tynkkynen LK, Sovala M, Jormanainen V, Keskimäki I. COVID-19 in Finland: Vaccination strategy as part of the wider governing of the pandemic. Health Policy and Technology. 2022;11(2).	Not Economic evaluation
Tomaiuolo R, Restelli U, Faggiano FC, Di Resta C, Al Bitar Nehme S, Giuliani F, et al. Health technology assessment to employ COVID-19 serological tests as companion diagnostics in the vaccination campaign against SARS-CoV-2. Clinical Chemistry and Laboratory Medicine. 2022;60(9):1463-77.	Not Economic evaluation
Towse A, Chalkidou K, Firth I, Kettler H, Silverman R. How Should the World Pay for a Coronavirus Disease (COVID-19) Vaccine? Value in Health. 2021;24(5):625-31.	Not Economic evaluation
Utami AM, Rendrayani F, Khoiry QA, Noviyanti D, Suwantika AA, Postma MJ, et al. Economic evaluation of COVID-19 vaccination: A systematic review. J. 2023;13:06001.	Wrong study design
Van der Pol S, Postma MJ, Boersma C. HPR40 Cost-Effectiveness and Budget Impact of Baloxavir Marboxil in the Netherlands Based on Post-COVID Influenza Season Scenarios. Value in Health. 2022;25(12):S239-S40.	Wrong Population
Vardavas C, Zisis K, Nikitara K, Lagou I, Aslanoglou K, Athanasakis K, et al. The cost of the COVID-19 pandemic vs the cost-effectiveness of mitigation strategies in the EU/UK/EEA and OECD countries: a systematic review. 2022.	Wrong study design
Vásquez WF, Trudeau JM, Alicea-Planas J. Immediate and informative feedback during a pandemic: Using stated preference analysis to predict vaccine uptake rates. Health Economics (United Kingdom). 2021;30(12):3123-37.	Wrong study design
Walkowiak MP, Walkowiak D. Am I Paid Well Enough to Be Diagnosed with COVID-19? Determinants of Gender Differences in Infection Detection Rate among Polish Working Age Population. Journal of Personalized Medicine. 2022;12(5).	Not Economic evaluation
Walkowiak MP, Walkowiak JB, Walkowiak D. COVID-19 passport as a factor determining the success of national vaccination campaigns: Does it work? the case of lithuania vs. Poland. Vaccines. 2021;9(12).	Not Economic evaluation
Wang WC, Fann JCY, Chang RE, Jeng YC, Hsu CY, Chen HH, et al. Economic evaluation for mass vaccination against COVID-19. Journal of the Formosan Medical Association. 2021;120:S95-S105.	Wrong Population

Xu FF, Brodszky V. HTA65 Socio-Economic Determinants of Health Status During COVID-19 Pandemic in Hungary. Value in Health. 2022;25(12):S309.	Not Economic evaluation
Yasri S, Wiwanitkit V. New COVID-19 vaccine: What about cost and utility? International Journal of Preventive Medicine. 2022;13(1):42.	Wrong Population
Yegorov S, Kadyrova I, Negmetzhanov B, Kolesnikova Y, Kolesnichenko S, Korshukov I, et al. Sputnik-V reactogenicity and immunogenicity in the blood and mucosa: A prospective cohort study. 2022.	Not Economic evaluation
Yu YC, Song Y. Etiology, clinical features, infection control and therapy of SARS-CoV-2 Omicron variant. Medical Journal of Chinese People's Liberation Army. 2022;47(11):1063-72.	Wrong Publication text
Zhao J, Fu Y, Han P, Yang L. SA6 A Review of Economic Evaluation on Vaccination and Non-Pharmaceutical Interventions for COVID-19 Prevention and Control. Value in Health. 2022;25(7):S605.	Not Economic evaluation
Zivin JG, ers N. The spread of COVID-19 shows the importance of policy coordination. Proceedings of the National Academy of Sciences of the United States of America. 2020;117(52):32842-4.	Not Economic evaluation

Studies with high risk of bias

Relevant articles but excluded after quality assessment due to high risk of bias.

Study
Pilz S, Ioannidis JPA. Does natural and hybrid immunity obviate the need for frequent vaccine boosters against SARS-CoV-2 in the endemic phase? European Journal of Clinical Investigation. 2023;53(2).
Volodymyrovych et al. Pharmaco Economics Analysis of COVID-19 Vaccines in Ukraine. Journal of Pharmaceutical Research International. 2021; 33(32A)

Appendix 3.

SBU Checklist applied to the four included studies

Checklist for assessing the quality of health economic modelling studies

**Author**	**Year**	**Article number**	**Reviewer, date**
**Pilz S, Ioannidis JPA**	**2023**		Tanto, 24-March-2023

	**High**	**Moderate**	**Low**	**Insufficient**	**Comments**
Assessment of the transferability of the study's economic results (Section 2):			Low		The assessment purely based on simulation
Assessment of the study quality with respect to economic aspects (Sections 3 and 4):			Low		The assessment purely based on simulation
Assessment of the study quality with respect to the effects and side effects of the intervention (assessed by the project experts):				Insufficient	Lack of clear information regarding the outcome and model projection and no adverse event information

**1. Study relevance (PICO) in relation to the project research questions** *For the study to be included, these questions must be answered by "yes".*	**Yes**	**No**	**Unclear**	**Not applicable**	**Comments**
a) Is the study population relevant?	Yes				The population is age-group based, however the data is all model projections only (not RWE)

b) Is the intervention relevant?	Yes				COVID-19 vaccination, however assumptions are made especially for vaccine efficacy
c) Is the comparator relevant?	Yes				Non-vaccinated group is based on projection/model
d) Is the outcome measure relevant?	Yes				It is NNT
**2. Transferability of the study's economic results**	**Yes**	**No**	**Unclear**	**Not applicable**	**Comments**
a) Are both costs and effects studied (or are the effects assumed to be equal)?	Yes				Effects splitted based on vaccine efficacy percentage, and cost is based on scnario of prices
b) Is the intervention implemented in a sector or by an organisation (e.g. hospital care or a local social service office) relevant to the current Swedish context?	Yes				Same vaccination strategy, and vaccine type and dosages
c) Are the unit costs used in the study relevant to the current Swedish context? [1]	Yes				It uses US dollar
d) Do the extent and the type of care or intervention delivered to study participants correspond to what patients/users receive in the current Swedish context?	Yes				Same vaccination strategy, and vaccine type and dosages
e) Does the study have a societal perspective?		No			Only health care perspective
**3. Potential conflicts of interest**	**Yes**	**No**	**Unclear**	**Not applicable**	**Comments**
a) Is there a low risk that the conflicts of interest declared by the authors may have influenced the study results?		No			Stated in the article
b) Is there a low risk that a sponsor with an economic interest in the outcome may have influenced the study results?		No			No external funding is declared

c) Is there a low risk of conflict of interest from other sources (e.g. the authors have developed the intervention)?		No			Not mentioned in the article
**4. Quality of the economic analysis**<br>**4.1 Choice of analysis**	**Yes**	**No**	**Unclear**	**Not applicable**	**Comments**
a) Is the type of economic analysis justified in relation to the research questions?	Yes				It is straight forward EE assessment, however all based on model/estimation only
**4.2 Model structure**	**Yes**	**No**	**Unclear**	**Not applicable**	**Comments**
a) Is the model structure appropriate for the specific research question and the specific health condition?			Unclear		The calculation is based on IFR, and assumptions/projections
b) Is the model structure, including the underlying assumptions, transparent?	Yes				The author elaborated clearly in the article, for instance the assumptions that all population is infected
c) Is the external validity of the model explored? [2]		No			Not mention in the article if there is any other studies similar to the one in the article
d) Is the time horizon sufficient to reflect all important differences in costs and effects?			Unclear		It is not mentioned in the article
e) Markov models: Is the model cycle length motivated by the research question?				Not applicable	
**4.3 Costs and effects**	**Yes**	**No**	**Unclear**	**Not applicable**	**Comments**
a) Have all relevant outcomes been identified (including side effects)?		No			The author considers the COVID-19 impact for death only

b) Is the data on treatment effects taken from the best possible sources? [3]		No			It is simulation
c) Is the difference in treatment effects, which determines the model outcomes, statistically significant?			Unclear		It is simulation
d) Are appropriate methods used to extrapolate treatment effects over the chosen time horizon? [4]			Unclear		It is simulation
e) Has the study considered compliance? [5]		No			Anti vaccine is not considered
f) Are the quality-of-life weights from the best possible sources?		No			It is simulation
g) Given the perspective of the analysis, have all relevant costs been identified (including those due to side effects)?		No			No side efect information is included in the article
h) Is the data on resource use (e.g. number of social worker visits, number of hospital care days) from the best possible sources?		No			It is simulation
i) Are the unit costs from the best possible sources?		No			It is simulation
**4.4 Interpretation of results**	**Yes**	**No**	**Unclear**	**Not applicable**	**Comments**
a) Was an incremental analysis of both costs and outcomes conducted (or is it possible to calculate)?			Unclear		Table 3 (purely simulation)
b) Are appropriate statistical methods used?			Unclear		No clear information on the calculation of the simulation
c) Are the conclusions consistent with the reported results?	Yes				See discussion section
**4.5 Sensitivity analysis**	**Yes**	**No**	**Unclear**	**Not applicable**	**Comments**
a) Are all important variables explored in sensitivity analyses? [6]				Not applicable	Not conducted

b) Is the uncertainty in the result explored using probabilistic sensitivity analysis?				Not applicable	Not conducted
c) Is the result insensitive to changes in examined variables? [7]				Not applicable	Not conducted
**4.6 Discounting (for studies with a time horizon exceeding 1 year) [8]**	**Yes**	**No**	**Unclear**	**Not applicable**	**Comments**
a) Are costs discounted appropriately?		No			Simulation does not consider the discount
b) Are outcomes discounted appropriately?		No			Simulation does not consider the discount

Checklist for assessing the quality of health economic modelling studies | **Reviewer, date**

**Author**	**Year**	**Article number**	**Reviewer, date**
**Orlewska K, Wierzba W, Sliwczynski A**	**2022**		Tanto, 28/03/2023

	**High**	**Moderate**	**Low**	**Insufficient**	**Comments**
Assessment of the transferability of the study's economic results (Section 2):		Moderate			Similar time period, population structure, type and doses of vaccine given and countries geography (affecting transmission), although must be considered that other measures were not implemented strictly in Sweden (ex. Social restrictions, etc)
Assessment of the study quality with respect to economic aspects (Sections 3 and 4):	High				Clear data and taken from the national registry (official data)
Assessment of the study quality with respect to the effects and side effects of the intervention (assessed by the project experts):		Moderate			Straight to the point with the data and explanation about the calculation, however to re-conduct this in Sweden, full study must be explored

**1. Study relevance (PICO) in relation to the project research questions**<br>*For the study to be included, these questions must be answered by "yes".*	**Yes**	**No**	**Unclear**	**Not applicable**	**Comments**

a) Is the study population relevant?	Yes				COVID-19 vaccinated population, based on age-group
b) Is the intervention relevant?	Yes				Limited to Pfizer vaccine, which available in Poland during 2021
c) Is the comparator relevant?	Yes				There is age-group based no vaacinated group
d) Is the outcome measure relevant?	Yes				QALY is used
**2. Transferability of the study's economic results**	**Yes**	**No**	**Unclear**	**Not applicable**	**Comments**
a) Are both costs and effects studied (or are the effects assumed to be equal)?	Yes				ICER can be calculated
b) Is the intervention implemented in a sector or by an organisation (e.g. hospital care or a local social service office) relevant to the current Swedish context?	Yes				It is official program by the government
c) Are the unit costs used in the study relevant to the current Swedish context? [1]	Yes				Since the treatment is similar, the result is transferable to Swedish circumstances
d) Do the extent and the type of care or intervention delivered to study participants correspond to what patients/users receive in the current Swedish context?	Yes				Population in Sweden, mostly receive Pfizer vaccine and minimum 2 dosages
e) Does the study have a societal perspective?		No			Strictly health care perspective, although mentioned in the deiscussion that including societal could potentially increase cost effectivenss of vaccination

3. Potential conflicts of interest	Yes	No	Unclear	Not applicable	Comments
a) Is there a low risk that the conflicts of interest declared by the authors may have influenced the study results?		No			Stated in the article
b) Is there a low risk that a sponsor with an economic interest in the outcome may have influenced the study results?			Unclear		Not mention in the article if there is any sponsor involved
c) Is there a low risk of conflict of interest from other sources (e.g. the authors have developed the intervention)?	Yes				One of the author works for Polish's ministry
**4. Quality of the economic analysis**<br><br>**4.1 Choice of analysis**	**Yes**	**No**	**Unclear**	**Not applicable**	**Comments**
a) Is the type of economic analysis justified in relation to the research questions?	Yes				
**4.2 Model structure**	**Yes**	**No**	**Unclear**	**Not applicable**	**Comments**
a) Is the model structure appropriate for the specific research question and the specific health condition?	Yes				It considers common pathway of disease and cost related to answer the research question
b) Is the model structure, including the underlying assumptions, transparent?	Yes				The author elaborated clearly in the article, for instance the assumptions that no re-infection in the calculation/model
c) Is the external validity of the model explored? [2]	Yes				It is mentioned in the discussion regarding results from other studies
d) Is the time horizon sufficient to reflect all important differences in costs and effects?	Yes				Clearly mention in the article the basis for choosing time period

e) Markov models: Is the model cycle length motivated by the research question?	Yes				The time period is given for each states based on disease progression references
**4.3 Costs and effects**	**Yes**	**No**	**Unclear**	**Not applicable**	**Comments**
a) Have all relevant outcomes been identified (including side effects)?	Yes				Although it is not calculated, for example long term COVID effect, however it is mentioned in the article
b) Is the data on treatment effects taken from the best possible sources? [3]	Yes				Data is taken from the national registry
c) Is the difference in treatment effects, which determines the model outcomes, statistically significant?	Yes				It is clear cut for state of disease progression
d) Are appropriate methods used to extrapolate treatment effects over the chosen time horizon? [4]	Yes				Over the chosen time period , yes, however the study is cut off for 1 year data only
e) Has the study considered compliance? [5]		No			Anti vaccine is not considered
f) Are the quality-of-life weights from the best possible sources?	Yes				Data is taken from the national registry
g) Given the perspective of the analysis, have all relevant costs been identified (including those due to side effects)?		No			No side efect information is included in the article
h) Is the data on resource use (e.g. number of social worker visits, number of hospital care days) from the best possible sources?	Yes				Data is taken from the national registry
i) Are the unit costs from the best possible sources?	Yes				Data is taken from the national registry

**4.4 Interpretation of results**	**Yes**	**No**	**Unclear**	**Not applicable**	**Comments**
a) Was an incremental analysis of both costs and outcomes conducted (or is it possible to calculate)?	Yes				Table 2 and table 3
b) Are appropriate statistical methods used?	Yes				In also includes lower and upper limit, and sensitivity analysis
c) Are the conclusions consistent with the reported results?	Yes				
**4.5 Sensitivity analysis**	**Yes**	**No**	**Unclear**	**Not applicable**	**Comments**
a) Are all important variables explored in sensitivity analyses? [6]	Yes				Page 1025, variables chosen for the DSA
b) Is the uncertainty in the result explored using probabilistic sensitivity analysis?		No			Only DSA
c) Is the result insensitive to changes in examined variables? [7]		No			Table 4, population >80, worst scenario result is the only one under ICER threshold
**4.6 Discounting (for studies with a time horizon exceeding 1 year) [8]**	**Yes**	**No**	**Unclear**	**Not applicable**	**Comments**
a) Are costs discounted appropriately?	Yes				No discount on the cost
b) Are outcomes discounted appropriately?	Yes				3.5% for the health outcome

Checklist for assessing the quality of health economic modelling studies **Reviewer, date**

Author **Year** **Article number** Tanto, 29-March-2023

Debrabant K, Grønbæk L, Kronborg C. **2021**

	**High**	**Moderate**	**Low**	**Insufficient**	**Comments**
Assessment of the transferability of the study's economic results (Section 2):	High				Geographically near, and also the same health care system as Sweden
Assessment of the study quality with respect to economic aspects (Sections 3 and 4):		Moderate			Data is clearly provided
Assessment of the study quality with respect to the effects and side effects of the intervention (assessed by the project experts):		Moderate			Side effect of the intervention is not elaborated, however the data is clear and limitation is explained

**1. Study relevance (PICO) in relation to the project research questions**<br>*For the study to be included, these questions must be answered by "yes".*	**Yes**	**No**	**Unclear**	**Not applicable**	**Comments**
a) Is the study population relevant?	Yes				The population is divided by age-group and also by scenario of percentage vaccination coverage
b) Is the intervention relevant?	Yes				
c) Is the comparator relevant?	Yes				Non-vaccinated group, and also other groups in different screnarios

d) Is the outcome measure relevant?	Yes				QALY is used
**2. Transferability of the study's economic results**	**Yes**	**No**	**Unclear**	**Not applicable**	**Comments**
a) Are both costs and effects studied (or are the effects assumed to be equal)?	Yes				From the description in the article, it is stated, outcome is not equal between scenarios
b) Is the intervention implemented in a sector or by an organisation (e.g. hospital care or a local social service office) relevant to the current Swedish context?	Yes				It is responsibility from the Danish government, same in Sweden, it is handled by government ministry
c) Are the unit costs used in the study relevant to the current Swedish context? [1]	Yes				It uses local currency, convertion to SEK is possible
d) Do the extent and the type of care or intervention delivered to study participants correspond to what patients/users receive in the current Swedish context?	Yes				It is similar with Sweden prioritization strategy
e) Does the study have a societal perspective?	Yes				It states as focusing to healthcare perspective, and also there is scenario involving productivity loss (limited societal perspective)
**3. Potential conflicts of interest**	**Yes**	**No**	**Unclear**	**Not applicable**	**Comments**
a) Is there a low risk that the conflicts of interest declared by the authors may have influenced the study results?		No			Stated in the article
b) Is there a low risk that a sponsor with an economic interest in the outcome may have influenced the study results?		No			Stated in the article

c) Is there a low risk of conflict of interest from other sources (e.g. the authors have developed the intervention)?		No			Stated in the article
**4. Quality of the economic analysis**<br>**4.1 Choice of analysis**	**Yes**	**No**	**Unclear**	**Not applicable**	**Comments**
a) Is the type of economic analysis justified in relation to the research questions?	Yes				The concept is valid
**4.2 Model structure**	**Yes**	**No**	**Unclear**	**Not applicable**	**Comments**
a) Is the model structure appropriate for the specific research question and the specific health condition?	Yes				Yes, it follows the disease progression pathways, also includes test + FU visit
b) Is the model structure, including the underlying assumptions, transparent?	Yes				It is mentioned in the articles several assumptions
c) Is the external validity of the model explored? [2]	Yes				It is mentioned as peer-reviewed, and comparison with similar EE in USA is elaborated
d) Is the time horizon sufficient to reflect all important differences in costs and effects?	Yes				Based on the data provided, it seems sufficient, although no reason is given why only 6 months
e) Markov models: Is the model cycle length motivated by the research question?				Not applicable	
**4.3 Costs and effects**	**Yes**	**No**	**Unclear**	**Not applicable**	**Comments**

a) Have all relevant outcomes been identified (including side effects)?		No			The authors consider treatment options for COVID-19 infection in the cost analysis, but not AE from vaccination itself
b) Is the data on treatment effects taken from the best possible sources? [3]	Yes				Data is taken from the official government registry
c) Is the difference in treatment effects, which determines the model outcomes, statistically significant?	Yes				Based on assumption that vaccine is 100% effective
d) Are appropriate methods used to extrapolate treatment effects over the chosen time horizon? [4]	Yes				Information on how to calculate the diagnosis and test required (p. 979)
e) Has the study considered compliance? [5]		No			Anti vaccine is not considered
f) Are the quality-of-life weights from the best possible sources?	Yes				Data is taken from the official government registry
g) Given the perspective of the analysis, have all relevant costs been identified (including those due to side effects)?		No			It focuses on health care perspective, but no AE cost is included
h) Is the data on resource use (e.g. number of social worker visits, number of hospital care days) from the best possible sources?	Yes				Data is taken from the official government registry
i) Are the unit costs from the best possible sources?	Yes				Data is taken from the official government registry
**4.4 Interpretation of results**	**Yes**	**No**	**Unclear**	**Not applicable**	**Comments**
a) Was an incremental analysis of both costs and outcomes conducted (or is it possible to calculate)?	Yes				Data is provided
b) Are appropriate statistical methods used?	Yes				Figure 4 is the key

c) Are the conclusions consistent with the reported results?	Yes				Discussion elaborates the result in detail
**4.5 Sensitivity analysis**	**Yes**	**No**	**Unclear**	**Not applicable**	**Comments**
a) Are all important variables explored in sensitivity analyses? [6]	Yes				Vaccine prices, vaccine efficacy stands at 100%, no co-morbidity, and productivity loss
b) Is the uncertainty in the result explored using probabilistic sensitivity analysis?			Unclear		It is not mentioned in the article
c) Is the result insensitive to changes in examined variables? [7]		No			Productivity loss, and in some degree, vaccine price (300DKK, figure 4B)
**4.6 Discounting (for studies with a time horizon exceeding 1 year) [8]**	**Yes**	**No**	**Unclear**	**Not applicable**	**Comments**
a) Are costs discounted appropriately?		No			No information for discount of cost
b) Are outcomes discounted appropriately?	Yes				It follows Ministry of Finance, Denmark for recommendation, however it also uses 2%, is there a possibility for this program to extend beyond 35 years?

Checklist for assessing the quality of health economic modelling studies **Reviewer, date**

**Author**	**Year**	**Article number**	Tanto, 29-March-2023
**Volodymyrovych et al.**	**2021**		

	**High**	**Moderate**	**Low**	**Insufficient**	**Comments**
Assessment of the transferability of the study's economic results (Section 2):			Low		Not enough detail for this study to be replicated
Assessment of the study quality with respect to economic aspects (Sections 3 and 4):				Insufficient	No essential data is provided to justify the result
Assessment of the study quality with respect to the effects and side effects of the intervention (assessed by the project experts):			Low		Many loop holes and weakness in the article, question arise for the 'peer-reviewed' status

**1. Study relevance (PICO) in relation to the project research questions** *For the study to be included, these questions must be answered by "yes".*	**Yes**	**No**	**Unclear**	**Not applicable**	**Comments**
a) Is the study population relevant?	Yes				The population is divided by age-group and also by scenario of percentage vaccination coverage
b) Is the intervention relevant?	Yes				The vaccine stated in the article is Pfizer, however for sensitivity analysis, other brands are included
c) Is the comparator relevant?	Yes				Non-vaccinated group
d) Is the outcome measure relevant?	Yes				QALY is used

2. Transferability of the study's economic results	Yes	No	Unclear	Not applicable	Comments
a) Are both costs and effects studied (or are the effects assumed to be equal)?	Yes				From the description in the article, it is stated, however details are poorly elaborated
b) Is the intervention implemented in a sector or by an organisation (e.g. hospital care or a local social service office) relevant to the current Swedish context?	Yes				It is responsibility from the Ukraine government, same in Sweden, it is handled by government ministry
c) Are the unit costs used in the study relevant to the current Swedish context? [1]	Yes				It uses local currency, convertion to SEK is possible
d) Do the extent and the type of care or intervention delivered to study participants correspond to what patients/users receive in the current Swedish context?	Yes				It is similar with Sweden prioritization strategy
e) Does the study have a societal perspective?			Unclear		It states as focusing to healthcare perspective, but in the same paragraph, it states also relation to societal. Ambiguous.
**3. Potential conflicts of interest**	**Yes**	**No**	**Unclear**	**Not applicable**	**Comments**
a) Is there a low risk that the conflicts of interest declared by the authors may have influenced the study results?		No			Stated in the article
b) Is there a low risk that a sponsor with an economic interest in the outcome may have influenced the study results?		No			Stated in the article
c) Is there a low risk of conflict of interest from other sources (e.g. the authors have developed the intervention)?		No			Stated in the article
**4. Quality of the economic analysis**	**Yes**	**No**	**Unclear**	**Not applicable**	**Comments**

4.1 Choice of analysis					
a) Is the type of economic analysis justified in relation to the research questions?	Yes				The concept is valid, however the details are poorly elaborated
**4.2 Model structure**	**Yes**	**No**	**Unclear**	**Not applicable**	**Comments**
a) Is the model structure appropriate for the specific research question and the specific health condition?			Unclear		It is not informative enough to learn the full concept idea. It is definitely not Markov.
b) Is the model structure, including the underlying assumptions, transparent?			Unclear		It is mentioned in the articles several assumptions, although not all is clear, example: section 2.2.1 regarding decreasing rate for 0-4 years old. What is the basis to lower it to 45?
c) Is the external validity of the model explored? [2]			Unclear		It is mentioned as peer-reviewed, however many contradictive information in the article.
d) Is the time horizon sufficient to reflect all important differences in costs and effects?			Unclear		Not mention in the article regarding the reason for timeframe (Apr-May 2021)
e) Markov models: Is the model cycle length motivated by the research question?				Not applicable	
**4.3 Costs and effects**	**Yes**	**No**	**Unclear**	**Not applicable**	**Comments**

a) Have all relevant outcomes been identified (including side effects)?		No			The authors consider treatment options for COVID-19 infection in the cost analysis, but not AE from vaccination itself
b) Is the data on treatment effects taken from the best possible sources? [3]	Yes				Data is taken from the official government registry
c) Is the difference in treatment effects, which determines the model outcomes, statistically significant?			Unclear		No details for this information, only summary in the article
d) Are appropriate methods used to extrapolate treatment effects over the chosen time horizon? [4]			Unclear		No information is provided
e) Has the study considered compliance? [5]		No			Anti vaccine is not considered
f) Are the quality-of-life weights from the best possible sources?	Yes				Data is taken from the official government registry
g) Given the perspective of the analysis, have all relevant costs been identified (including those due to side effects)?		No			It focuses on health care perspective, but no AE cost is included
h) Is the data on resource use (e.g. number of social worker visits, number of hospital care days) from the best possible sources?	Yes				Data is taken from the official government registry
i) Are the unit costs from the best possible sources?	Yes				Data is taken from the official government registry
**4.4 Interpretation of results**	**Yes**	**No**	**Unclear**	**Not applicable**	**Comments**
a) Was an incremental analysis of both costs and outcomes conducted (or is it possible to calculate)?		No			No data is provided, example QALy data is not available
b) Are appropriate statistical methods used?			Unclear		No information is provided

c) Are the conclusions consistent with the reported results?			Unclear		Not enough information in the result section to justify discussion section
**4.5 Sensitivity analysis**	**Yes**	**No**	**Unclear**	**Not applicable**	**Comments**
a) Are all important variables explored in sensitivity analyses? [6]			Unclear		It is stated in the article, however the calculation is not provided, example: lower-higher limit information is not available
b) Is the uncertainty in the result explored using probabilistic sensitivity analysis?			Unclear		Sensitivity analysis is mentioned as 'conducted', however no detail data regarding it.
c) Is the result insensitive to changes in examined variables? [7]			Unclear		Sensitivity analysis is mentioned as 'conducted', however no detail data regarding it.
**4.6 Discounting (for studies with a time horizon exceeding 1 year) [8]**	**Yes**	**No**	**Unclear**	**Not applicable**	**Comments**
a) Are costs discounted appropriately?			Unclear		No information for discount of cost
b) Are outcomes discounted appropriately?			Unclear		Contradictive information between abstract and section 2.4

[1] Provided that they, if necessary, are converted to Swedish krona [SEK], and adjusted to the current price year according to purchasing power parity (PPP). The following cost converter is used: http://eppi.ioe.ac.uk/costconversion/default.aspx

[2] External validity involves comparing the outcomes of the model with those from other models or empirical studies. It may also involve having the model peer reviewed. A mere comparison of the study's incremental cost-effectiveness ratio (ICER) with that of other studies is not sufficient for a “yes” answer.

[3] Are there other studies or studies of better quality that contain data on the effects of the intervention that should have been included in the analysis? If there are several high quality studies, are the results synthesized in a meta-analysis?

[4] Are assumptions regarding a sustained treatment effect after the follow-up period clearly presented and discussed?

[5] Has the study considered compliance, possibly supplemented with information on whether analyses were performed according to intention-to-treat (ITT)? Do patients/users and care providers employ the intervention as intended (e.g. the number of sessions in a treatment programme)?

[6] Concerns variables containing uncertainty that may influence the results of the analysis. If extrapolations are made from empirical data, it may be important to explore different methods of extrapolating.

[7] Concerns the robustness of the results, i.e. that the sensitivity analyses do not alter the overall conclusions about cost-effectiveness (regarding both one-way and probabilistic sensitivity analysis).

Internal

[8] Is the selected approach justified? Different countries have different recommendations. Future costs should be discounted (but the discount rate may vary). For future outcomes, there are arguments both for and against discounting. In Sweden, the Dental and Pharmaceutical Benefits Agency recommends a discount rate of 3% for both costs and effects, but also requires sensitivity analyses with rates of 0 and 5%.

Appendix 4

Data Extraction Table

General study characteristics	Orlewska,2022	Debrabant,2021
First author and year of publication	Orlewska,2022	Debrabant, 2021
Sources of funding	Not stated	Stated,<br>University of Southern Denmark (employer)
Competing interests	Stated,<br>There are no conflicts of interests.	Stated,<br>There are no conflicts of interests.
Setting	Poland	Denmark
Patient characteristics	5 groups: general population in Poland, and based on age-group (30-39, 40-49, 60-69 and >80)	Population is based on the two age-groups (18-60 years old and 60 years old and older) and divided into 4 scenarios:<br>1. Vaccination to 25 percent of total Danish population who are 60 years old and older.<br>2. Vaccination to 25 percent of total Danish population who are 18-60 years old.<br>3. Vaccination to 15 percent of total Danish population who are 18-60 years old and 25 percent of total Danish population who are 60 years old and older.

		4. Vaccination to 40 percent of total Danish population who are 18-60 years old.
Type of intervention	Pfizer Comirnaty vaccine	COVID-19 vaccination with assumption of 95% efficacy (no specific brand is mentioned)
Control treatment	5 groups: unvaccinated general population in Poland, and based on age-group (30-39, 40-49, 60-69 and >80)	Control is only one group which is non-vaccinated Danish general population.
Eligibility criteria	General population in Poland, and divided by 4 age-group (30-39, 40-49, 60-69 and >80)	Danish population who are 18 years old and older.
Methods and outcomes of economic evaluations		
Time frame of the analysis (time horizon)	1 year	6 months
If model based	Markov	Dynamic transmission model
Data source of resource use	Polish Agency for Health Technology Assessment and Tariff System (AOTMiT)	- deleted entry on a social media platform by a European Minister of Health (vaccine's price)<br>- the regional health authorities (vaccine administration cost)<br>- The Danish Health Data Authority (DHDA): hospitalization cost<br>- a telephone survey by Statens Serum Institut: productivity loss<br>- Statistics Denmark: productivity loss<br>- statistics from Eurostat: productivity loss

Assumptions of the measurement of resources		
Costs (in reported currency or in converted currency)	Year 2020, in PLN (Polish Zloty).<br>Exchange rate: 1 euro = 4.44 PLN	Year 2020, in DKK (Danish Krona)<br>Exchange rate: 1 euro = 7.4 DKK
Data source of effects	- mortality: life years lost with survival age data from Statistics Poland<br>- QALY: questionnaire from other study<br>- vaccine efficacy: Pfizer clinical data	- life-years: administrative data from Statistics Denmark and Staten Serum Institut<br>- QALY: other study (55)<br>- Ministry of Finance Denmark for discount rate
Methods of measurement of effects	willingness-to-pay-threshold, which in Poland is 3 × GDP/per capita (147,024 PLN/QALY gained in 2020)	no specific threshold, instead the result is compared with other preventive programmes in Denmark
Incremental cost–effectiveness ratios	Base case: Vaccination to 60-69 and >80 is cost saving. While cost effective for other age-group and in general population.<br><br>ICER (PLN/QALY):<br>- general population: 6,249<br>- age 30-39: 67,823<br>- age 40-49: 28,135<br>- age 60-69: cost saving<br>- >80: cost saving	Scenarios 2 and 4 are dominated thus it is no longer included in evaluation.<br><br>ICER base-case for scenario 1 is between 53,000-118,000 DKK/QALY (vaccine price 300-500 DKK), and scenario 3 is between 319,000-803,000 DKK/QALY (vaccine price 300-500 DKK).
Outcome(s) of analyses of sensitivity analyses	In the general population and in age-group 30-39ICER is most sensitive to the vaccine effectiveness, vaccine price, and SARS-CoV-2 infection rates.	Cost of hospitalisation does not affect ICER, however mortality rate and vaccine efficacy do.<br><br>Vaccine price and inclusion of productivity loss affect cost effectiveness of the program.

Authors' conclusions	In most scenarios, vaccination to population 60-69 and >80, is cost saving, and in general and to in? all other age-groups, vaccination is cost effective.	Without productivity loss, the elderly population should always be part of the target group for a COVID-19 vaccination programme and compared to other programmes, it is cost effective.<br><br>Taking productivity loss into account, at least in the case of low vaccine prices (300 DKK), vaccinating the younger population first can be cost effective (key is fig 4).

Appendix 5

Calculation of COVID-19 vaccination effect in Sweden based on Orlewska's data

A	B	C	D	E	F	G	H	I	J	K
Age-group	Based on Persson, U. et al. (39) – unvaccinated scenario			Number of expected deaths per 1000 individuals' base case (based on Orlewska) (26)		Simulation of the situation in Sweden if COVID-19 vaccination program is implemented				Orlewska data (26)
	QALYs lost per premature death	Number of deaths based on excess mortality in Sweden	Total QALY-loss due to excess mortality 2020	No vaccination program	With vaccination program	Number of deaths based on excess mortality in Sweden (vaccinated)	Difference of deaths from vaccinated and non-vaccinated scenario	Total QALY-loss due to excess mortality 2020 (vaccinated)	Gained QALY per avoidable death if vaccinated	Orlewska QALY lost/death
30-39	20	23	455.59	0.07	0.01	1.85	21.15	37.25	19.78	19.87
40-49	18	54	963.27	0.20	0.02	4.59	49.41	83.12	17.81	17.09
60-69	12.09	494	5,882.87	1.75	0.14	39.24	454.76	474.38	11.89	10.43
>80	4.06	4,705	19,089.30	11.86	0.97	383.62	4,321.38	1,556.44	4.06	2.12
General population	5.98	7,049	42,159.97	2.28	0.18	556.50	6,492.50	3,328.42	5.98	7.54

The explanation for the calculation:

- Person, U. et al. (39) provided the data of non-vaccinated Swedish population (column B, C, D).
- Orlewska (26) showed the base case expected deaths per 1000 individuals in non-vaccinated (column E) and in vaccinated scenario (column F).
- Number of death based on excess mortality in Sweden (in vaccinated scenario) (Column G) is calculated from column C multiplied with column F and divided by column E. (G = (CxF) / E)
- Column H (Difference of death cases from vaccinated and non-vaccinated scenario) is calculated from Number of deaths based on excess mortality in Sweden (column C) subtracted with Number of deaths based on excess mortality in Sweden (vaccinated) (Column G). (H = C - G)
- Total QALY-loss due to excess mortality 2020 (vaccinated) (Column I) is calculated from QALYs lost per premature death (Column B) multiplied with Number of deaths based on excess mortality in Sweden (vaccinated) (Column G) (I = B x G)
- Lost QALY per avoidable death if vaccinated (Column J) is calculated from (Total QALY-loss due to excess mortality 2020 (Column D) - Total QALY-loss due to excess mortality 2020 (vaccinated) (Column I) divided with Difference of death cases from vaccinated and non-vaccinated scenario (Column H). (I = (D-I) / H).
